# Exceedance Probability and Toxicity-Value Sensitivity in Health-Risk Assessment of a Pb–Zn Slag Reprocessing Site Under the 2025 IRIS Arsenic Values

**DOI:** 10.3390/toxics14100894

**Published:** 2026-10-07

**Authors:** Zhaksylyk Pernebayev, Akmaral Issayeva, Togzhan Baiduisenova, Mahmood-ur-Rahman Ansari, Akbota Aitimbetova, Kural Nurseiit

**Affiliations:** 1 LLP Centre for Scientific Research and Ecological Expertise KazEcoHolding, Shymkent 160012, Kazakhstan; pernebayev.zh@kazecoholding.kz (Z.P.); a.aitimbetova@kazecoholding.kz (A.A.); k.nurseit1507@mail.ru (K.N.); 2 Department of Biology, Zhanibekov University, Shymkent 160012, Kazakhstan; 3 Department of Bioinformatics and Biotechnology, Government College University Faisalabad, Faisalabad 38000, Pakistan; mahmoodansari@gcuf.edu.pk

**Keywords:** probabilistic risk assessment, Monte Carlo simulation, exceedance probability, toxicity value revision, arsenic, sensitivity analysis, Central Asia

## Abstract

Screening-level risk assessments report single-point estimates of carcinogenic risk (CR) and hazard index (HI), conveying neither the probability of exceedance nor its sensitivity to revised toxicity values. We examined both at an active lead–zinc slag reprocessing site in Shymkent, Kazakhstan, by reanalysing a published dataset (24 soil samples) with Monte Carlo simulation for an adult outdoor worker, under the toxicity values of the source assessment and under the January 2025 IRIS values for inorganic arsenic. Under the original values, the probability of exceeding CR = 10^−4^ was 70% (95% bootstrap interval 59–80%) and of exceeding HI = 1 was 82%. Applied to the same concentrations, the 2025 values gave a 21-fold higher mean risk and exceedance probabilities of 99% and 97%. The screening conclusion therefore holds under both sets of values, whereas the quantitative estimate does not. Arsenic concentration dominated output variance. Because ingestion and dermal contact carry over 99% of the worker’s risk, risk reduction requires measures that interrupt these pathways: engineered capping of the heap perimeter, restricted access, and occupational hygiene. Phytostabilization gave no modelled benefit to on-site workers; its value lies in limiting off-site dust, which was not modelled. Arsenic bioaccessibility testing and denser sampling are priorities.

## 1. Introduction

Reprocessing of historical lead–zinc (Pb–Zn) slag occupies an uneasy position in the circular-economy agenda. Secondary recovery of residual metals diverts material from permanent disposal and reduces demand for primary extraction, yet the same operations disturb consolidated waste bodies and can remobilise legacy contaminants where containment, dust suppression and runoff control are inadequate [1,2,3,4], a pattern documented across non-ferrous smelting sites worldwide [5]. Few regions in Central Asia carry a Pb–Zn metallurgical legacy on the scale of southern Kazakhstan, and the slag heap of the former Shymkent lead plant—accumulated over roughly seven decades of pyrometallurgical operation and currently under active reprocessing—is among the most severely contaminated sites documented in the region [6,7,8]. Decisions about such sites are increasingly framed as sustainability trade-offs: how much intervention is warranted, where, and on what evidence.

The evidence base for site-management decisions is predominantly deterministic. Assessments typically report single-point estimates of carcinogenic risk (CR) and hazard index (HI), computed from mean or maximum measured concentrations combined with default exposure factors, and compare these against regulatory thresholds. Such an estimate answers whether a threshold is exceeded by the calculated value. It does not answer how probable exceedance is across the range of plausible exposures at the site, which is the quantity a manager weighing intervention against cost requires. Probabilistic methods, for which formal guidance exists within the same regulatory framework [9], supply that quantity by propagating distributions rather than point values through the exposure model, and by permitting the contribution of each input to output variance to be ranked so that data-collection effort can be directed where it reduces uncertainty most [10,11]. Applications in mining and smelter settings are now well established: probabilistic assessments have been reported for soils around Pb/Zn smelters [12], for mining-area soils with Monte Carlo simulation coupled to an improved exposure model [13], and for age-stratified receptor groups (a receptor being the person or population group whose exposure is modelled) where exceedance probabilities were reported separately by population subgroup [11,14], and for source-resolved assessment across a linked water–soil–crop system [15]. A smaller number of studies have applied deterministic and probabilistic models to the same dataset; one such comparison, across 444 paired soil–crop samples, reported that conventional deterministic methods underestimate risk for the most exposed 15–25% of the population [10]. Adoption in the post-Soviet Central Asian context, however, appears limited relative to the scale of the metallurgical legacy there.

A second and less-examined source of uncertainty is the toxicity values themselves. Slope factors and reference doses are periodically revised as epidemiological evidence accumulates, and a revision can move a risk estimate by more than the spatial variability the assessment was designed to characterize. In January 2025 the US EPA revised its assessment of inorganic arsenic, raising the oral slope factor from 1.5 to 32 per mg·kg^−1^·d^−1^ on the basis of a Bayesian hierarchical dose–response meta-analysis of bladder and lung cancer endpoints, and lowering the oral reference dose; the inhalation unit risk was not revised [16]. The revision has now been in force for over a year and a half, during which the stock of assessments of arsenic-dominated sites completed under the superseded values has remained in use without recomputation; such assessments are not directly comparable with those completed after the revision, and the site-management decisions resting on them have not been revisited. The practical consequences of the transition have been addressed in regulatory guidance and in the consultancy literature, chiefly through recomputation of generic screening levels, and agency guidance on applying the revised cancer slope factor was issued in 2026 [17]. Whether the site-specific management conclusions of completed assessments survive the revision is a different question, answerable only site by site, and has not, to the authors’ knowledge, been examined in the peer-reviewed literature for Pb–Zn metallurgical legacy sites.

Arsenic is a particularly consequential case. It combines a high slope factor with a tendency toward spatially discrete enrichment at smelter and slag sites, so that the risk estimate for an arsenic-dominated location is effectively a function of a single toxicity value applied to a strongly skewed concentration distribution. Both components of that product were affected by the 2025 revision: the revised oral slope factor of 32 per mg·kg^−1^·d^−1^ was obtained by combining endpoint-specific values for bladder (17.6) and lung (21.3) cancer [16], and the oral reference dose was lowered to 6.0 × 10^−5^ mg·kg^−1^·d^−1^ on the basis of ischemic heart disease and type 2 diabetes endpoints [16]. Where arsenic dominates a site’s risk profile, the sensitivity of that profile to the revision is correspondingly large and cannot be inferred without recomputation.

**Figure 1:**
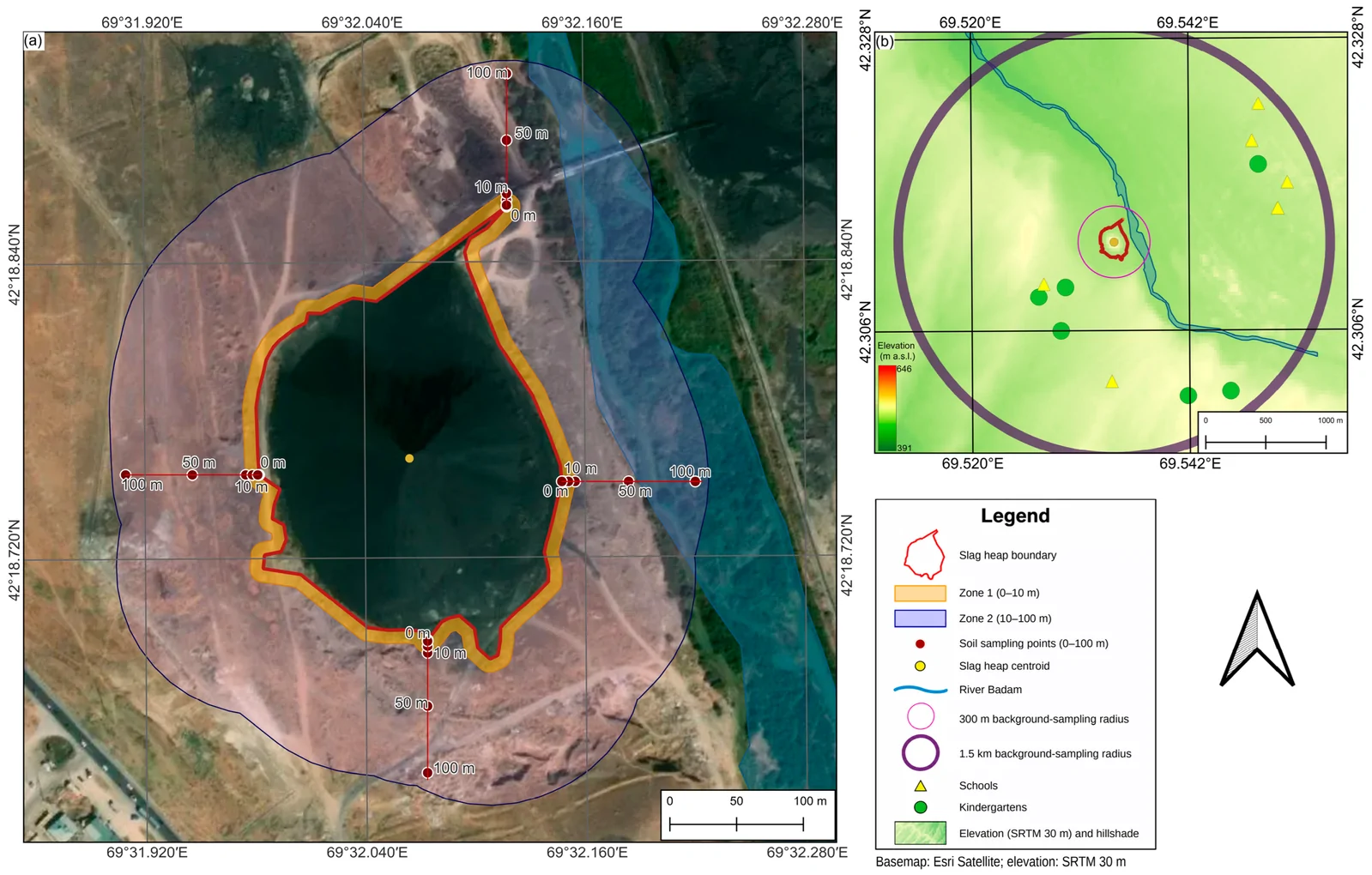
Study site. (**a**) Slag heap boundary and centroid (42.3125° N, 69.5342° E), the four sampling transects with points at 0, 1, 5, 10, 50 and 100 m from the heap edge (points at 1 and 5 m are not labelled), exposure Zone 1 (0–10 m) and Zone 2 (10–100 m), and the Badam River. (**b**) Surroundings of the heap: elevation (m a.s.l.) from the SRTM 30 m digital elevation model with hillshade, the Badam River, schools and kindergartens, and circles of 300 m and 1.5 km radius marking the distances at which the two reference samples were collected. Basemap: Esri Satellite.

Both questions are addressed here at an active Pb–Zn slag reprocessing site in Shymkent, Southern Kazakhstan, using the geochemical dataset published in [8]. The source assessment reported deterministic CR and HI estimates for an adult occupational receptor together with a three-tier remediation zonation. The same receptor is used here: an adult outdoor worker on the site. Children, residents of the adjacent districts (Figure 1b) and other potentially sensitive groups are not modelled, and the results should not be read as applying to them. This scope bears most directly on the conclusions about phytostabilization and off-site dust transport, which concern exposure pathways and populations beyond the site worker (Section 4.5). The present paper draws on that published dataset rather than on new field sampling; its contribution is the probabilistic and toxicity-value-sensitivity framework applied to it, and the general result that framework yields—that at arsenic-dominated sites the qualitative management conclusion and the quantitative risk estimate have different robustness properties, the former surviving both input variability and a toxicity-value revision while the latter survives neither. The same 24 transect samples are reanalysed probabilistically under two parameterizations. The first retains the toxicity values applied in the source assessment, so that the two analyses remain directly comparable; the second uses the values currently in effect. Exceedance probabilities, sensitivity rankings and scenario projections are reported under each.

Because the present analysis reuses a recently published dataset, its relation to the source assessment is set out explicitly in Table 1. Pernebayev et al. [8] reported the geochemistry, pollution indices, plant biomonitoring, ecosystem risk and deterministic human-health estimates for the site, and identified a probabilistic reanalysis of the human-health component as a planned follow-up. None of those results is repeated here. The present study carries out the probabilistic reanalysis with the data available, extends it to the toxicity values currently in force, quantifies the uncertainty attached to the result, and tests the zonation and intervention priorities proposed in [8] against the risk distributions.

The specific objectives were to: (i) Fit and validate concentration distributions for eight priority metals; (ii) quantify CR and HI as full probability distributions and report exceedance probabilities against the 10^−6^, 10^−4^ and HI = 1 screening levels; (iii) locate the previously published point estimates within those distributions; (iv) repeat the analysis under the 2025 IRIS arsenic values and determine which conclusions are altered; (v) rank the contribution of each stochastic input to output variance; and (vi) evaluate the published zonation and project risk under alternative intervention scenarios. Although developed at one site, the framework and its findings apply to any assessment in which a single strongly skewed contaminant governs the risk profile, and the procedure for testing whether a management conclusion survives a toxicity-value revision is transferable to the wider stock of arsenic-dominated assessments completed before January 2025.

## 2. Materials and Methods

### 2.1. Study Area

The study site is an active Pb–Zn slag reprocessing facility in Shymkent, Southern Kazakhstan (centre of the slag heap: 42.3125° N, 69.5342° E). The surrounding Turkestan Region is semi-arid (Köppen BSk [18]), receiving roughly 400 mm of precipitation annually; easterly and westerly winds prevail and govern which directions dust from the heap travels. The Badam River flows along the eastern side of the heap, between the heap and the former lead-smelter site; the A-2 highway and residential areas lie to the west. A detailed description of site geology, hydrogeology, slag mineralogy (SEM-EDS: PbS, ZnS, ZnO, PbO, Fe_2_O_3_; slag pH 7.5–8.5) is given in Pernebayev et al. [8].

The heap lies on a terrace of the Badam River valley at the foot of dissected piedmont slopes (Figure 1b), so that surface runoff from these slopes during spring snowmelt and rainfall events is directed across the site toward the river. The zonal soils of the piedmont belt of the Turkestan Region are loamy sierozems [19]; around the heap, however, soils are disturbed by slag reprocessing and vehicle traffic. Soil agrochemical and water-physical properties, slope gradients and erosion rates were not measured in the source campaign and are not available for the present reanalysis. The nearest residential buildings lie about 250 m west of the heap boundary. Gridded population estimates (WorldPop, 2020, constrained and adjusted to United Nations estimates, 100 m resolution [20]) indicate approximately 1300 residents within 500 m of the heap centre and 10 500 within 1 km; one school (about 570 m) and three kindergartens (about 480–750 m) lie within 1 km of the heap boundary (Figure 1b).

### 2.2. Data Source

No new field sampling was conducted for this study. The geochemical dataset comprises 24 transect soil samples and two distal reference samples collected on 24 April 2026 and published as Supplementary Table S1 of [8]. Sampling followed GOST R 58595-2019 [21], GOST 17.4.3.01-2017 [22] and GOST 17.4.4.02-2017 [23]; samples were collected at a 0–50 cm depth along four transects oriented to the cardinal points, sampled at 0, 1, 5, 10, 50 and 100 m from the heap perimeter. Reference samples were collected at 300 m and 1.5 km. Two of the six authors of the present paper (Z.P. and A.A.) are authors of [8]; the present work is therefore in part a reassessment by the original investigators of their own published dataset, undertaken to test whether the management conclusions drawn there survive treatment of input variability and the subsequent revision of arsenic toxicity values. The dataset is published under a CC BY licence and the analysis reported here can be reproduced independently from it using the supplied code.

At each sampling point a composite sample of approximately 1 kg was formed from five increments collected by the envelope method (GOST 17.4.4.02-2017 [23]) over the 0–50 cm layer with a steel shovel, and placed in a polyethylene bag. Each point is therefore represented by a single depth-integrated composite; no depth-resolved data were obtained. Samples were transported in insulated cool boxes and were received by the laboratory on the day of collection (24 April 2026), as recorded in the test protocols. According to METAR records from the Shymkent airport station (ICAO: UAII) [24], light showers fell during the night and morning of 23 April 2026 and ended by about 10:00 local time; 24 April was dry and clear, with light winds of 1–4 m·s^−1^ and relative humidity falling from about 90% before dawn to about 30% in the afternoon. The samples were delivered unprocessed, and preparation before acid digestion was carried out by the laboratory. The heap, the four transects, Zones 1 and 2 and the Badam River are shown in Figure 1a.

The 0–50 cm interval is deeper than the surface layer normally considered for incidental ingestion and dermal contact. It was retained because the receptor is a worker at an active reprocessing site, where excavation, loading and vehicle traffic bring soil from below the immediate surface into contact with workers, so that exposure is not confined to the uppermost centimetres. Because the samples are depth-integrated, concentrations in the surface layer may be higher or lower than the values modelled here. This uncertainty was bounded by scaling all concentrations by 0.5 and 2.0 (Table S3): under the 2025 IRIS values P(CR > 10^−4^) remained between 97.8% and 99.8% across this range, and under the source-assessment values it ranged from 53.1% to 83.4%.

Ten elements—As, Cd, Cr, Cu, Fe, Hg, Mn, Ni, Pb and Zn—were determined by ICP-OES (Shimadzu ICPE-9000) after acid digestion, in accordance with method M 02-902-157-10, at the accredited Analytical Laboratory of LLP KazEcoAnaliz (Almaty, Kazakhstan; accreditation certificate KZ.T.02.1017, issued 25 February 2026). Analyses were performed 27 April–5 May 2026 under Order No. 23-26 (Test Protocols No. 23-01 and No. 23-02, dated 5 May 2026). Quality control was carried out under the laboratory’s internal procedures within the scope of its accreditation. The test protocols issued to the authors report the results, method, instrument and analytical conditions (25.2–25.4 °C; relative humidity 54–55%), but do not include batch-level quality-control records; element-specific recoveries of certified reference materials, blank values and replicate statistics were therefore not available for the present reanalysis. None of the 24 transect samples returned a value below the reporting limit for any of the eight modelled metals: the only soil reporting limit stated in the protocols, 0.1 mg·kg^−1^ for Hg, lies below the lowest transect Hg value (0.12 mg·kg^−1^), so no substitution of censored values was required. To bound the effect of analytical error on the modelled distributions, measured concentrations were (i) scaled uniformly by 0.8 and 1.2 and (ii) perturbed by random multiplicative errors of 10% and 20% relative standard deviation before refitting (200 replicates each); results are given in Table S3.

Eight metals were carried forward into the risk model: Pb, Zn, Cd, Cu, As, Hg, Ni and Cr. The model is centred on arsenic, but the other seven metals were retained because carcinogenic risk and the hazard index are, by definition, sums across all contaminants of potential concern, and because the dominance of arsenic is a result to be demonstrated rather than assumed. Retaining them allows the contribution of each metal to total CR and HI to be quantified (Section 3.5). Fe and Mn were not treated as contaminants of potential concern: Fe concentrations (104–9570 mg·kg^−1^) were below its upper-crustal abundance (39 200 mg·kg^−1^ [8]) at every transect point, and Mn (61–358 mg·kg^−1^) did not exceed the Kazakhstan MPC at any point.

For the zonal analysis (Section 2.6), samples were assigned to two zones following the three-tier framework proposed in [8]: Zone 1, 0–10 m (*n* = 16) and Zone 2, 10–100 m (*n* = 8). The third tier (>100 m) contains no transect samples and was not modelled.

### 2.3. Concentration Distributions

Lognormal distributions were fitted to each metal by maximum likelihood on log-transformed concentrations across the 24 transect samples. The lognormal assumption was tested by the Shapiro–Wilk statistic applied to the log-transformed values (α = 0.05). With 24 observations per element the test has limited power, so non-rejection is treated as consistency with the lognormal model rather than as confirmation of it; normal quantile–quantile plots for all eight metals are provided as Figure S1 in the Supplementary Material. As a further check, lognormal, gamma and Weibull distributions were fitted by maximum likelihood to each metal and compared by the Akaike information criterion (AIC) and the Kolmogorov–Smirnov test, and the full risk analysis was repeated with each alternative model (Section 3.1; Table S4).

Six of the eight metals were consistent with a lognormal model (Table 2). Zn (W-test *p* < 0.001) and Hg (*p* < 0.001) were not. Hg exhibits a bimodal distribution—37.9–47.4 mg·kg^−1^ at four of the six western-transect points against 0.12–0.98 mg·kg^−1^ at seventeen of the remaining eighteen—consistent with the two distinct depositional processes identified in [8], and its geometric standard deviation (6.31) is more than 1.5× that of any other element. For these two metals, concentrations were resampled empirically from the measured values with a multiplicative lognormal kernel (σ = 0.15) to avoid imposing a parametric form the data do not support. A parametric model was therefore used where the Shapiro–Wilk test on log values did not reject lognormality (*p* ≥ 0.05) and empirical resampling where it did. The kernel smooths the 24 discrete measured values so that simulated concentrations are not restricted to exactly those values; σ = 0.15 is about one tenth of the standard deviation of the log-transformed concentrations (1.26 for Zn and 1.84 for Hg), so it does not alter the shape of the distributions. Because Zn and Hg together contribute about 0.3% of the mean hazard index, results are insensitive to this choice: for σ between 0 and 0.30 no reported probability changed by more than 0.1 percentage point (Table S4).

Sample 260 (eastern transect, 100 m), which returned anomalously low Fe and Zn in the source study, was retained: it was excluded from enrichment-factor calculations in [8] on normalization grounds that do not apply to the present analysis.

### 2.4. Probabilistic Risk Model

Human-health risk was modelled for an adult outdoor-worker receptor only following the US EPA RAGS framework [25], with three exposure pathways: incidental soil ingestion, inhalation of resuspended particulates and dermal contact. Chronic daily intake (CDI, mg·kg^−1^·d^−1^) was calculated for each pathway as in [8], preserving the model structure so that the two analyses differ only in the treatment of input variability.

**Figure 2:**
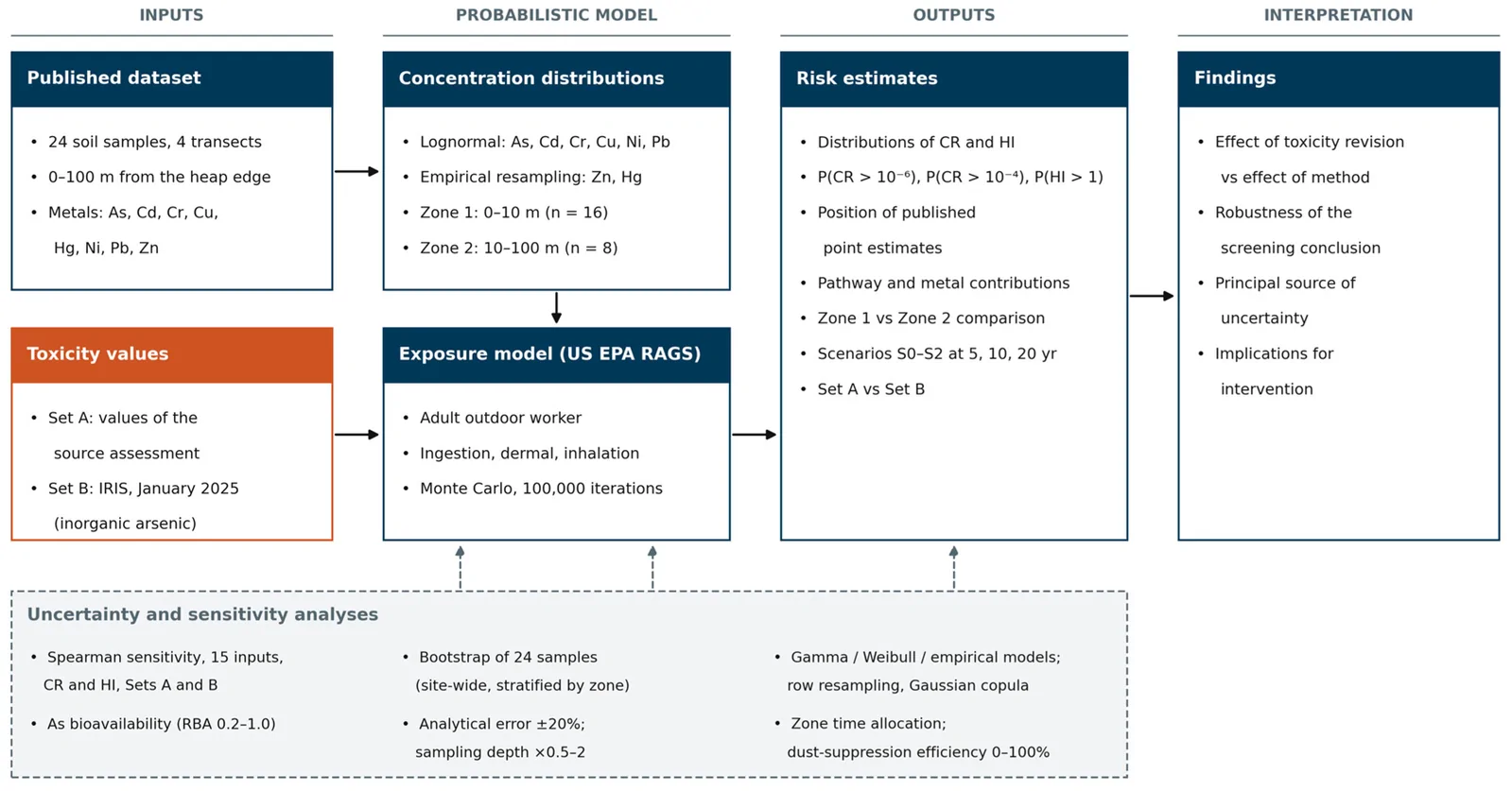
Workflow of the analysis. The published dataset of [8] is used to fit concentration distributions, which are propagated through the US EPA RAGS exposure model for an adult outdoor worker under two sets of toxicity values. Uncertainty and sensitivity analyses (bottom, dashed arrows) act on the concentration distributions, the exposure model and the outputs.

Three sources of uncertainty are distinguished and treated separately. Variability—the spatial variation in concentrations across the site and inter-individual variation in exposure factors—is represented by the input distributions and propagated by the Monte Carlo simulation; this is what the exceedance probabilities describe. Parameter uncertainty—the uncertainty in the fitted distributions themselves, arising from the small number of samples and from the choice of distributional model—is quantified by bootstrap resampling and by the alternative concentration models described in Section 2.3 and below. Toxicity-value uncertainty is treated not probabilistically but as a comparison between two parameterizations (Section Toxicity Values), because the question it addresses is whether the conclusions survive a specific revision rather than how uncertain a given value is. The three are reported separately and are not combined into a single interval. The workflow of the analysis is summarized in Figure 2.

Exposure parameters were represented as distributions rather than point values (Table 3). Central values follow the US EPA Exposure Factors Handbook [26] and match the deterministic defaults used in [8], so that any divergence in output reflects distributional treatment alone and not a change in central assumptions.

Dermal absorption factors were metal-specific: As 0.030, Pb 0.006, and 0.001 for Zn, Cd, Cu, Hg, Ni and Cr. Following RAGS Part E, the dermal dose was expressed as an absorbed dose before multiplication by the slope factor, and the oral slope factor was adjusted to an absorbed-dose basis using a gastrointestinal absorption fraction of 1.0; absorption is therefore applied once and not twice.

Carcinogenic risk was computed as CR = Σ(CDI_i_ × SF_i_) across metals and pathways for As, Cd, Cr and Ni; non-carcinogenic hazard as HI = Σ(CDI_i_/RfD_i_) across all eight metals and three pathways. Slope factors and reference doses are listed in Table 4.

Results are interpreted against screening levels rather than binary thresholds. Following US EPA practice, carcinogenic risk is compared with the risk-management range of 10^−6^ to 10^−4^: 10^−6^ is the point of departure below which risk is generally not of concern, and 10^−4^ the upper end of the range, above which remedial action is generally warranted [27]; values within the range call for site-specific judgement. An HI above 1 indicates that the estimated intake exceeds the reference dose and signals that further evaluation is needed; it is not a prediction that adverse effects will occur. Because total arsenic, default exposure parameters and a single occupational receptor are used, all results are screening-level estimates, and ‘exceedance’ refers to these screening levels throughout.

In the primary analysis, concentrations of the eight metals were sampled independently from their marginal distributions. Because the measured samples are paired multi-element observations, the effect of this assumption was tested with two alternatives that preserve the observed dependence: joint resampling of complete sample rows, with and without the kernel described in Section 2.3, and a Gaussian copula combining the observed Spearman correlation matrix with the marginal distributions of Section 2.3 (Section 3.1; Table S4).

Arsenic risk was computed from total concentrations, which is equivalent to a relative oral bioavailability (RBA) of 1. Sensitivity to this assumption was examined by applying RBA values of 0.2, 0.4, 0.6 (the US EPA default for arsenic in soil [28]) and 0.8 to the ingestion pathway; dermal absorption is governed separately by the absorption factor, and the inhalation pathway was left unchanged (Section 3.3; Table S8).

Simulations were run with 100,000 iterations using the PCG64 generator (seed 20260827) in Python 3.11 with NumPy 2.4 [29] and SciPy 1.17 [30]. Convergence was verified by repeating the full analysis at 10 000 iterations under the same seed: exceedance probabilities agreed to within 0.9% and tail percentiles to within 5.1%, while the means of CR and HI agreed to within 1.6%. At 100,000 iterations the Monte Carlo standard error of an exceedance probability is at most 0.16 percentage points. This figure describes numerical precision conditional on the fitted distributions; it does not capture uncertainty arising from the limited number of measured samples, which is addressed below. The comparison is reproduced by the Supplementary Scripts.

Because the concentration distributions are fitted to 24 samples, their parameters are themselves uncertain. This uncertainty was quantified by nonparametric bootstrap. The 24 transect samples were resampled with replacement 1000 times; for each resample the lognormal parameters (As, Cd, Cr, Cu, Ni, Pb) were re-estimated and the empirical distributions (Zn, Hg) rebuilt, and the risk model was run with 10,000 iterations using the same exposure-parameter draws. The 2.5th and 97.5th percentiles across replicates are reported as 95% bootstrap intervals for the mean CR, mean HI and the exceedance probabilities. For the zonal comparison, a stratified bootstrap was applied, resampling Zone 1 (*n* = 16) and Zone 2 (*n* = 8) separately, and the ratio of mean CR between zones was recorded in each of 1000 replicates. With *n* = 24 these percentile intervals are approximate and indicate the order of magnitude of sampling uncertainty rather than exact coverage.

#### Toxicity Values

Set A comprises the toxicity values applied in the source assessment [8]. As a check on the present model implementation, the full deterministic model was evaluated at the RAGS point values on the measured concentrations, without recourse to any quantity reported in [8]. Under IRIS toxicity values throughout, site-mean carcinogenic risk is 6.44 × 10^−4^, 8.0% below the published figure of 7.0 × 10^−4^, and the worst-case sample yields 4.00 × 10^−3^ against a published 4.3 × 10^−3^. Adding an oral slope factor for cadmium of 6.1 per mg·kg^−1^·d^−1^—a value issued in several national frameworks but not by IRIS—raises the site-mean result to 7.02 × 10^−4^, within 0.3% of the published figure, the entire residual of 5.80 × 10^−5^ being attributable to that single term. The model structure is therefore reproduced; the element-wise apportionment differs solely through the treatment of cadmium described below, and all results reported here use IRIS values only. Any divergence between the deterministic and probabilistic results reported below reflects the treatment of input variability rather than a difference in model structure.

The oral and dermal slope factors applied to chromium are not issued by IRIS, which derives a carcinogenicity value for hexavalent chromium by the inhalation route only; the values used here were taken from national compilations and applied to total chromium, and chromium contributes 0.2% of total carcinogenic risk at this site. An inhalation RfC for inorganic arsenic has not been assessed under the IRIS programme. Route-specific inhalation and dermal reference doses used in the hazard index calculation were therefore derived by route extrapolation from published compilations rather than taken directly from IRIS, by applying a factor of 0.41 to the oral reference dose under either parameterization; this is noted in Section 4.6. Cadmium is classified by IRIS as carcinogenic by the inhalation route only, and no oral slope factor is issued for it; cadmium carcinogenicity is therefore restricted to the inhalation pathway throughout the present analysis, a pathway contributing less than 0.1% of intake under the RAGS default particulate emission factor. Assessments that apply an oral slope factor to cadmium, as some national frameworks do, will apportion a correspondingly larger share of total carcinogenic risk to that element.

### 2.5. Sensitivity Analysis

The contribution of each stochastic input to output variance was quantified by Spearman rank correlation between the sampled input values and each output—total CR and HI—across the 100,000 iterations, under both parameterizations. All fifteen stochastic inputs were included: the eight metal concentrations and the seven exposure parameters. An approximate contribution to output variance was obtained by normalizing the squared rank correlations to 100%. Rank correlation was preferred over Pearson correlation because the input–output relationships are monotonic but not linear, and because several inputs are strongly right-skewed.

### 2.6. Scenario Analysis

Risk trajectories were projected over 5-, 10- and 20-year horizons under three management scenarios, constructed by mechanism rather than by a uniform percentage reduction: no intervention in either zone (S0); engineered containment of Zone 1 (0–10 m), with no intervention in Zone 2 (S1); and engineered containment of Zone 1 combined with phytostabilization of Zone 2 (10–100 m) (S2).

Engineered containment was modelled as interruption of all three exposure pathways within the treated zone, consistent with regulatory guidance, which describes caps and covers as eliminating direct exposure to solid mining waste by creating a physical barrier and as reducing or eliminating fugitive dust emissions, rather than as producing a partial reduction in concentration [31]. An establishment lag of 1.0 yr was assumed for construction.

Phytostabilization was modelled as suppression of the inhalation pathway only, with ingestion and dermal contact retained, since a vegetative cover reduces dust emission without removing surface soil from contact. An establishment lag of 3.5 yr was applied, based on field data reporting 21–61% canopy cover after 41 months on compost-amended tailings [32]. The underlying flux basis is a documented reversal from net emission (2323 g·m^−2^·yr^−1^, unirrigated control) to net deposition (371 and 606 g·m^−2^·yr^−1^ at 16% and 32% canopy cover) [3]. These measurements were not converted into a fractional reduction in inhalation exposure; instead, the inhalation pathway was fully suppressed after the establishment lag, which represents the most favourable case for phytostabilization. Because the cited evidence indicates partial rather than complete cover, the dust-reduction efficiency was additionally varied from 0 to 100% in steps of 25%, so that the inhalation pathway was reduced by that fraction after the lag (Section 3.7; Table S6).

Both studies were conducted at the Iron King Mine and Humboldt Smelter Superfund site (Dewey-Humboldt, AZ, USA), on sulfidic mine tailings enriched in arsenic and lead under a semi-arid climate, where compost-amended plots were seeded with native plant species [3,32]. Canopy cover is the proportion of the ground surface covered by the vertical projection of plant foliage. The net emission or deposition flux is the difference between the mass of wind-blown particles leaving and arriving at a unit area of surface per year; a change from net emission to net deposition therefore indicates that the surface has ceased to act as a dust source. The cited setting is comparable to the Shymkent heap in climate and in the contaminants involved, which is why these data were used. It differs in substrate: the Shymkent slag is coarser (0.5–15 mm) and slightly alkaline (pH 7.5–8.5) [8], and no amendment or planting programme is in place at the site. Vegetation establishment on the slag may therefore be slower and less complete than in the cited trials, and the transferred values are treated as indicative rather than site-specific.

The projection horizon T governs the fraction of the exposure period over which an intervention is credited; it does not replace the exposure duration, which is sampled from its distribution as in Table 3. For each horizon T, the residual fraction of a suppressed pathway was computed as 1 − max(0, (T − lag)/T), i.e., the intervention is credited only for the portion of the exposure period during which it is operational.

Common random numbers were used across scenarios: concentrations were drawn once per zone and reused for all three scenarios, so that between-scenario differences reflect the modelled intervention alone and not simulation noise. Zonal results were combined assuming equal receptor time allocation between Zone 1 and Zone 2. In the absence of information on how workers divide their time, equal weighting was used as a neutral default; its influence was examined by repeating the scenario analysis for Zone 1:Zone 2 allocations of 100:0, 75:25, 25:75 and 0:100 (Section 3.7; Table S7).

### 2.7. Data and Code Availability

The source geochemical data are published as Supplementary Table S1 of [8]. Simulation code is provided as Supplementary Material and reproduces all reported values from the published dataset.

## 3. Results

### 3.1. Concentration Distributions

Fitted distribution parameters are given in Table 2 (Section 2.3). Geometric standard deviations ranged from 1.94 (Ni) to 6.31 (Hg), confirming substantial right-skew across all priority metals; for the four metals entering the carcinogenic-risk calculation they were 3.94 (As), 3.65 (Cd), 2.22 (Cr) and 1.94 (Ni). The ratio of arithmetic to geometric mean reaches 2.66 for As (1753 versus 658.2 mg·kg^−1^) and 2.40 for Cd (53.7 versus 22.4 mg·kg^−1^).

Six of the eight metals were consistent with a lognormal model (Shapiro–Wilk on log values, *p* = 0.108–0.955). Zn and Hg were not (*p* < 0.001 in both cases). The measured concentrations along the four transects, expressed as multiples of the Kazakhstan maximum permissible concentration, are mapped in Figure S2. Interpolated concentration surfaces were not produced: with four radial transects, the sectors between them are unsampled, and interpolation would assign values to large areas without measurements.

The lognormal model had the lowest AIC for five of the six parametrically modelled metals (for As, ΔAIC = 8.5 relative to the gamma and 6.2 relative to the Weibull model); for Ni the three models were indistinguishable (ΔAIC < 0.5), and no model was rejected by the Kolmogorov–Smirnov test (*p* ≥ 0.16; these *p* values are conservative because the parameters were estimated from the same data). Replacing the lognormal model with gamma or Weibull distributions, or using empirical resampling for all eight metals, changed P(CR > 10^−4^) from 69.9% to 67.7–71.9% under Set A and from 99.3% to 95.5–99.8% under Set B (Table S4). The lighter upper tails of the gamma and Weibull models reduce the Set B probabilities by about four percentage points; no conclusion is altered. Normal quantile–quantile plots of the log-transformed concentrations (Figure S1) provide a check that does not depend on test power, and the bootstrap gives 95% intervals for the fitted As parameters of 397–1115 mg·kg^−1^ for the geometric mean and 2.59–5.23 for the geometric standard deviation (Table S2).

Pb, Zn, Cd, Cu and Ni were strongly inter-correlated (Spearman ρ = 0.80–0.90), whereas As was weakly and non-significantly correlated with all metals except Hg (ρ = 0.17–0.32; As–Hg ρ = 0.47, *p* = 0.021). Because As dominates both CR and HI and is nearly independent of the other metals, preserving the dependence structure had little effect. Under Set A, P(CR > 10^−4^) was 68.1% with joint row resampling and 70.0% with the Gaussian copula, against 69.9% with independent sampling; under Set B it was 99.3–99.8% (99.3%). P(HI > 1) was 78.1–79.7% under Set A (82.1%) and 96.5–98.3% under Set B (97.3%) (Table S4). The independence assumption therefore does not materially affect the conclusions.

These departures have different causes. Mercury shows the largest dispersion of any element (GSD = 6.31, more than 1.5 times that of any other metal) and a clearly bimodal distribution: values of 37.9–47.4 mg·kg^−1^ at four of the six western-transect points against 0.12–0.98 mg·kg^−1^ at seventeen of the remaining eighteen, a separation of more than an order of magnitude with only three intermediate values (2.36 and 6.98 mg·kg^−1^ on the western transect and 2.44 mg·kg^−1^ elsewhere). This is consistent with the two distinct depositional processes identified in the source assessment [8] and is not adequately represented by any single unimodal parametric form. Zinc departs less dramatically but spans 7.79 to 2870 mg·kg^−1^, and its lowest value comes from sample 260, retained here for the reasons given in Section 2.3. For both metals, empirical resampling preserves the observed structure without imposing a form the data do not support; neither metal contributes materially to carcinogenic risk, so the choice affects the hazard index only. The two modes of Hg correspond to two depositional settings—the western transect and the other three—rather than to a mixture within a single population. Empirical resampling reproduces both modes in the observed proportions, whereas a two-component mixture or a transect-stratified model fitted to six and eighteen observations would be poorly constrained; and because Hg contributes about 0.3% of the mean hazard index, the choice of Hg model does not affect any reported result.

For the four metals entering the carcinogenic-risk calculation the lognormal fits were satisfactory, and arsenic—which dominates the output (Section 3.5)—returned a *p* value of 0.176, below those of Cr (0.955) and Ni (0.373) but above that of Cd (0.108). The As distribution spans 95.2 to 10,900 mg·kg^−1^ across the 24 transect samples, a range of more than two orders of magnitude within a 100 m radius, which is the feature that makes a point-estimate summary problematic at this site.

### 3.2. Distributions of Carcinogenic Risk and Hazard Index

**Figure 3:**
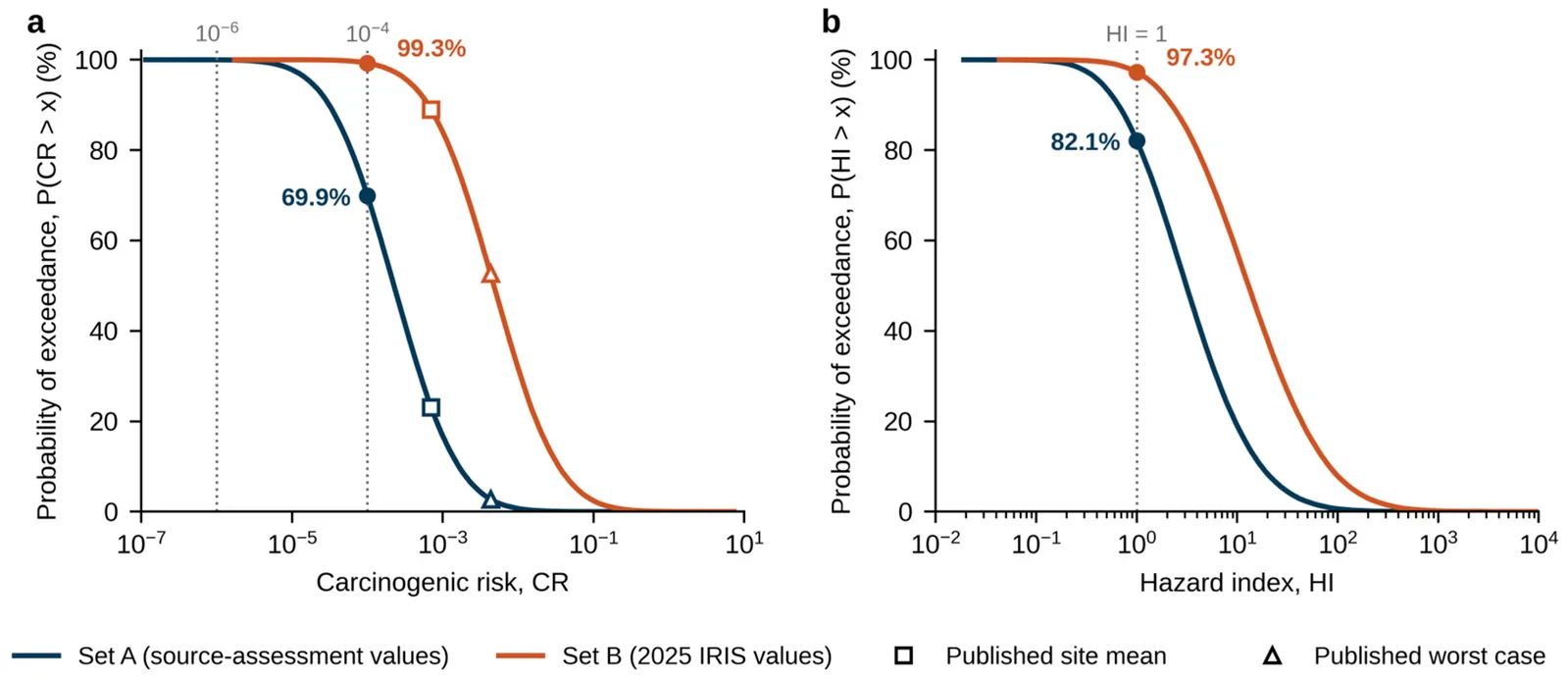
Exceedance curves for (**a**) total carcinogenic risk (CR) and (**b**) the hazard index (HI) across 100,000 Monte Carlo iterations, adult outdoor-worker scenario, under the toxicity values applied in the source assessment (Set A) and the values established by the January 2025 IRIS revision (Set B). Each curve gives the probability that the simulated value exceeds the level on the horizontal axis. Dotted lines mark 10^−6^ and 10^−4^, the lower and upper ends of the US EPA risk-management range, and HI = 1; filled circles give the probability of exceeding 10^−4^ and HI = 1. Open symbols mark the site-mean (7.0 × 10^−4^, squares) and worst-case (4.3 × 10^−3^, triangles) point estimates reported for the same dataset in [8]: under Set A these are exceeded by 23.1% and 2.8% of simulated exposures, and under Set B by 89.0% and 52.7%.

The distributions of CR and HI under both parameterizations are summarized in Table 5. Both are strongly right skewed under either parameterization. Under Set A the CR interquartile range spans 7.91 × 10^−5^ to 6.38 × 10^−4^ while the 99th percentile reaches 8.20 × 10^−3^—two orders of magnitude above the first quartile—and the mean (7.38 × 10^−4^) exceeds the median (2.26 × 10^−4^) by a factor of 3.3. A mean that sits above the 75th percentile is characteristic of a distribution whose expectation is governed by its upper tail rather than by its central mass. The same structure appears in the hazard index (mean 8.03, median 3.12) and is preserved under Set B, since a slope factor scales the distribution without altering its shape. Figure 3 shows both distributions as exceedance curves, which give the probability of exceeding any level of CR or HI. In the extreme upper tail the simulated values exceed unity under Set B (maximum 7.34), where the linear low-dose extrapolation underlying the slope-factor model no longer holds; such iterations should be read as an indication of the weight of the tail rather than as risk estimates in the probabilistic sense.

### 3.3. Exceedance Probabilities

Exceedance probabilities against the screening levels are given in Table 6. Under Set A, exceedance of the 10^−4^ carcinogenic screening level is more likely than not (69.9%) but far from certain, and exceedance of 10^−3^ occurs in roughly one simulated exposure in six. Under Set B both statements change character: exceedance of 10^−4^ becomes effectively certain (99.3%) and exceedance of 10^−3^ becomes the majority outcome (84.0%). The non-carcinogenic endpoint behaves similarly, with P(HI > 1) rising from 82.1% to 97.3% and P(HI > 10) from 19.1% to 57.4%.

The distinction matters for how a conclusion is stated. Under Set A the defensible statement is that risk is more likely than not to exceed the upper end of the risk-management range; under Set B, that it almost certainly does. Neither statement is available from a point estimate, and the two differ in the degree of confidence a manager could attach to a decision to intervene.

Sampling uncertainty is considerably larger than numerical uncertainty (Table S2). Across 1000 bootstrap replicates, P(CR > 10^−4^) ranged from 59% to 80% under Set A and from 98.4% to 99.9% under Set B (95% intervals), and P(HI > 1) from 72.5% to 90.1% and from 94.5% to 99.2%. In every replicate both exceedance probabilities remained above 50% under either parameterization.

Relative bioavailability of arsenic affects the Set A results more than the Set B results (Table S8). At the US EPA default RBA of 0.6, mean CR was 29% lower than with total arsenic, and P(CR > 10^−4^) was 62.5% under Set A and 98.7% under Set B; at an RBA of 0.2 the corresponding probabilities were 49.5% and 97.2%. The reduction in mean CR is less than proportional to RBA because dermal contact, which carries 28% of CR, is not affected. P(HI > 1) remained above 72% under Set A and above 93% under Set B across the range.

### 3.4. Position of the Published Point Estimates

The position of each published point estimate within the simulated distributions is given in Table 7. With the source-assessment values, 23.1% of simulated exposures exceed the published site-mean CR and 2.8% exceed the published worst case. Substituting the current toxicity values shifts both figures to 89.0% and 52.7%: what was reported as the site worst case now falls near the median of the distribution.

Mean CR under Set B is 21.3× that under Set A; mean HI is 4.7×. The ratio for CR follows directly from the 21.3-fold increase in the arsenic oral slope factor, since ingestion and dermal contact together account for 99.8% of arsenic CR (inhalation 0.17%). Because the concentrations and the exposure model are identical under both parameterizations, this difference reflects the toxicity values alone; it implies no change in the contamination of the site or in the exposure of the worker.

The effect of moving from deterministic to probabilistic treatment is small compared with the effect of the toxicity-value revision. Evaluated at the RAGS central-tendency exposure values and site-mean concentrations, the present model gives CR = 6.4 × 10^−4^ under Set A and 1.37 × 10^−2^ under Set B; the corresponding probabilistic means are 7.4 × 10^−4^ and 1.57 × 10^−2^, higher by a factor of 1.15 under either set. Replacing Set A with Set B multiplies CR by 21.2 in the deterministic and 21.3 in the probabilistic calculation. For HI the corresponding factors are 1.3 (probabilistic versus deterministic) and 4.7 (Set B versus Set A). The toxicity-value revision therefore moves the estimate by more than an order of magnitude, whereas the change in method moves it by 15–30%. What the probabilistic treatment adds is not a different central value but the distribution around it: the probability of exceeding 10^−4^ (69.9% under Set A, 99.3% under Set B) and HI = 1 (82.1% and 97.3%), and the position of the published point estimates within that distribution (Table 7). The published deterministic site-mean estimate of 7.0 × 10^−4^ [8] differs from the value reproduced here (6.4 × 10^−4^) because it included a legacy oral slope factor for cadmium (Section Toxicity Values).

### 3.5. Contaminant Contributions and Sensitivity

Arsenic accounted for 99.8% of mean CR under both parameterizations, with Cr contributing 0.2% and Cd and Ni together less than 0.05%. Arsenic also dominated the non-carcinogenic endpoint, contributing 91.7% of mean HI under Set A, followed by Pb (6.9%) and Cd (1.0%).

Sensitivity ranking (Spearman ρ with total CR; Table 8) is unchanged between the two parameterizations, since a slope factor scales the output without altering rank order.

**Figure 4:**
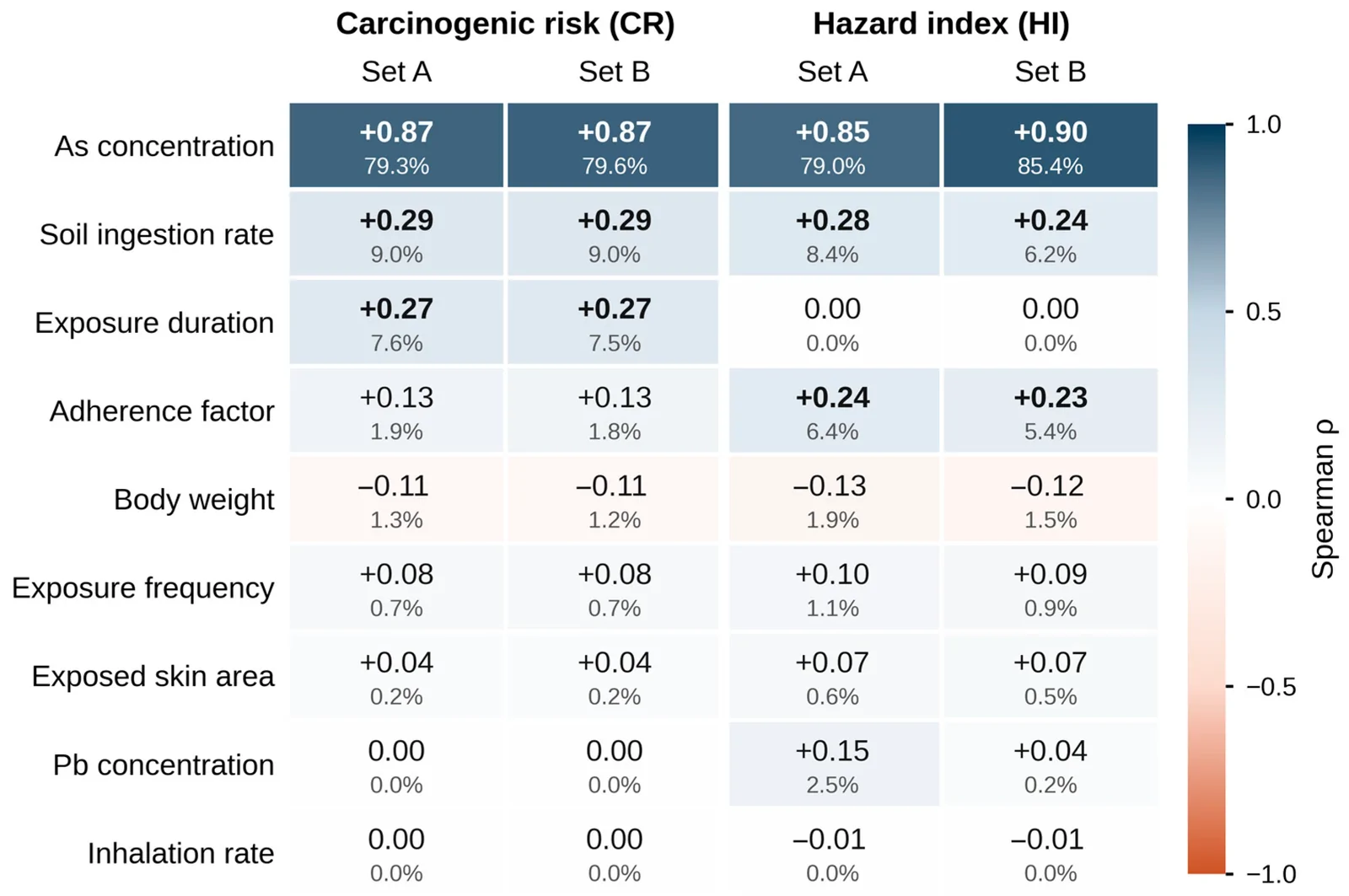
Sensitivity of total carcinogenic risk (CR) and the hazard index (HI) to the stochastic model inputs across 100,000 Monte Carlo iterations, under the source-assessment (Set A) and 2025 IRIS (Set B) toxicity values. Each cell gives the Spearman rank correlation between the input and the output (upper value; colour scale) and the approximate contribution of the input to output variance, obtained by normalizing the squared correlations of all fifteen inputs to 100% (lower value). Arsenic concentration dominates both outputs (ρ = +0.867 for CR under Set A), exceeding the next-ranked input by a factor of three. Exposure duration has no influence on HI, and Pb concentration is the only other metal with a detectable influence (HI, Set A). Concentrations of the remaining six metals (|ρ| ≤ 0.03) are not included in the figure; they are reported, together with the full ranking, in Table S5.

Arsenic concentration dominates, exceeding the next-ranked input by a factor of three (Figure 4).

With all fifteen stochastic inputs included, the concentrations of the seven metals other than As show negligible rank correlation with CR (|ρ| ≤ 0.005). For HI, As concentration again dominates (ρ = +0.852 under Set A and +0.904 under Set B, corresponding to about 79% and 85% of explained variance). The HI ranking differs from the CR ranking in two respects. Exposure duration has no influence on HI (ρ = −0.001), because for non-carcinogenic endpoints it cancels between intake and averaging time, whereas the adherence factor rises in rank (ρ = +0.242 under Set A) because dermal contact contributes 48% of mean HI against 28% of mean CR. Pb concentration is the only non-As metal with a detectable influence on HI (ρ = +0.153 under Set A, about 2.5% of explained variance; +0.044 under Set B). The full ranking for both outputs and parameterizations is given in Table S5.

Body weight carries the only negative correlation, as expected from its position in the denominator of the intake equation. The four exposure parameters that a site-specific occupational survey could realistically refine—exposure duration, ingestion rate, adherence factor and frequency—together rank well below arsenic concentration.

Inhalation rate shows negligible rank correlation (ρ < 0.01). This follows from the RAGS default particulate emission factor of 1.36 × 10^9^ m^3^·kg^−1^, under which the inhalation chronic daily intake is more than three orders of magnitude smaller than the ingestion intake; the inhalation pathway contributes 0.17% of arsenic carcinogenic risk under Set A (0.01% under Set B), against 71.4% for ingestion and 28.4% for dermal contact. This pathway structure governs the scenario results reported in Section 3.7.

### 3.6. Zonal Risk Distribution

**Figure 5:**
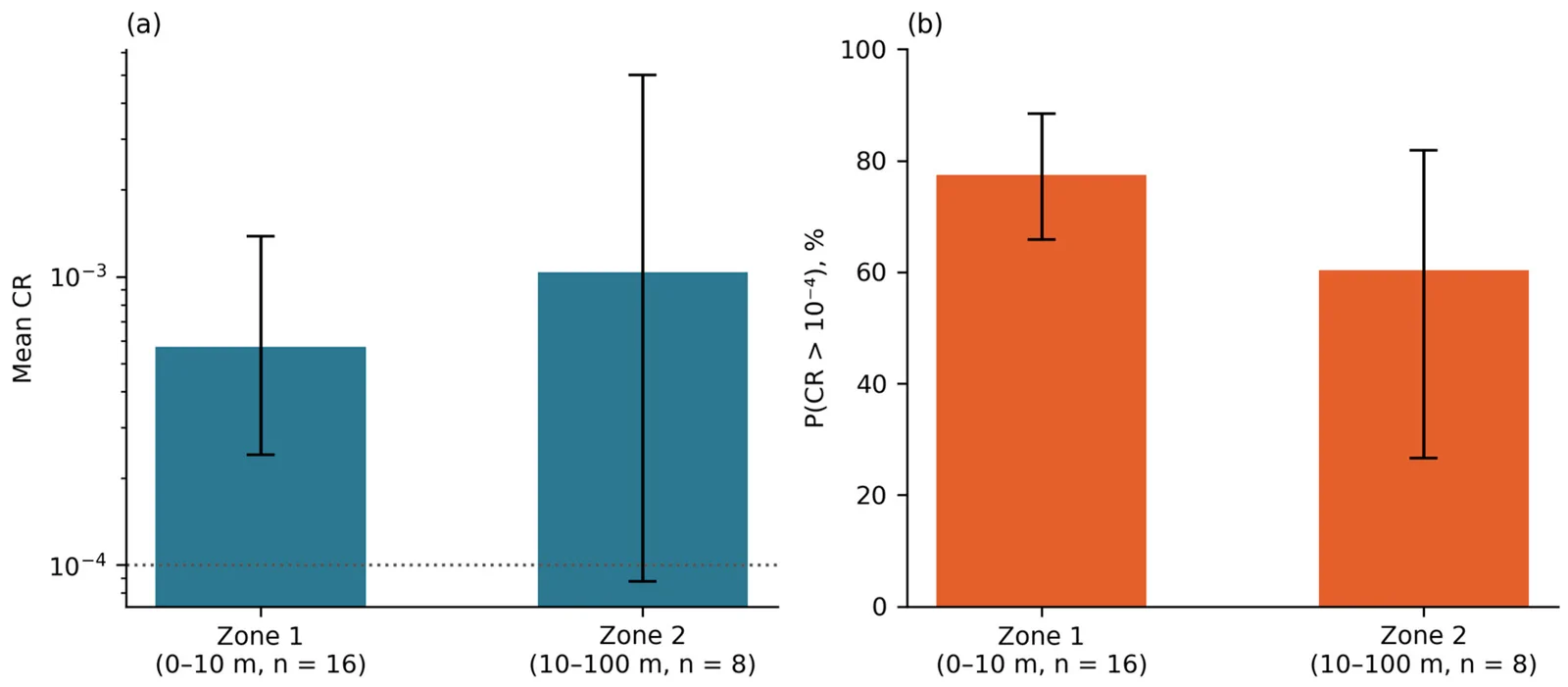
Zonal comparison at the 20-year horizon under the no-intervention scenario, Set A toxicity values: (**a**) Mean carcinogenic risk (logarithmic axis; the dotted line marks 10^−4^) and (**b**) probability of exceeding 10^−4^. Error bars show 95% stratified-bootstrap intervals (1000 replicates, zones resampled separately). Zone 2 has the higher point estimate of mean risk and Zone 1 the higher exceedance probability, but the intervals overlap widely and the zonal ordering is not resolved by the data (Section 3.6). The Zone 2 mean is carried by two of eight samples (Table 10).

Risk was computed separately for the two zones of the published three-tier framework (Table 9, Figure 5).

Mean risk in Zone 2 is 1.81× that in Zone 1, and mean HI is 1.72× higher. Zone 1 nevertheless carries the higher exceedance probability (77.5% versus 60.3%).

These two statements are not contradictory and describe different features of the distributions. Zone 1 is uniformly contaminated: nearly all sampling points exceed 10^−4^, but few reach extreme values. Zone 2 is spatially patchy: a majority of points exceed 10^−4^, but the distribution carries substantially heavier upper values, driven by the western-transect secondary maxima at 50 and 100 m (As 7610 and 7900 mg·kg^−1^; Pb 7850 mg·kg^−1^ at 100 m). Mean risk is governed by the tail; exceedance probability by the bulk.

The non-monotonic decay producing these secondary maxima was documented in [8], which reported that a simple radial dispersion model does not describe the contamination plume and declined to fit one. The consequence for remediation prioritization was not drawn there and is developed in Section 4.4.

Because the zonal comparison rests on eight outer-zone samples, its stability was tested by leave-one-out analysis on mean As concentration (Table 10).

Exclusion of either western-transect sample reverses the ordering; exclusion of any of the remaining six leaves the ratio at 1.49–1.56. The zonal comparison is therefore carried by two of eight samples, both on the transect identified in [8] as carrying the highest mean concentrations of Pb, Cd, Hg and As. The interpretation of this dependence is developed in Section 4.4.

Note that the Zone 2:Zone 1 ratio computed on mean As concentration (1.38) is lower than the ratio of mean modelled CR (1.81). The two are not directly comparable: risk is computed from fitted lognormal distributions, whose expectation is more sensitive to the upper tail than the arithmetic mean of the underlying samples. The larger ratio for risk than for mean concentration is the same tail effect described in Section 3.2, operating within rather than between frameworks.

The stratified bootstrap confirms that the zonal comparison is not resolved by the data: the 95% interval for the Zone 2:Zone 1 ratio of mean CR is 0.16–9.9, and Zone 2 exceeded Zone 1 in 68% of replicates.

### 3.7. Scenario Projections

**Figure 6:**
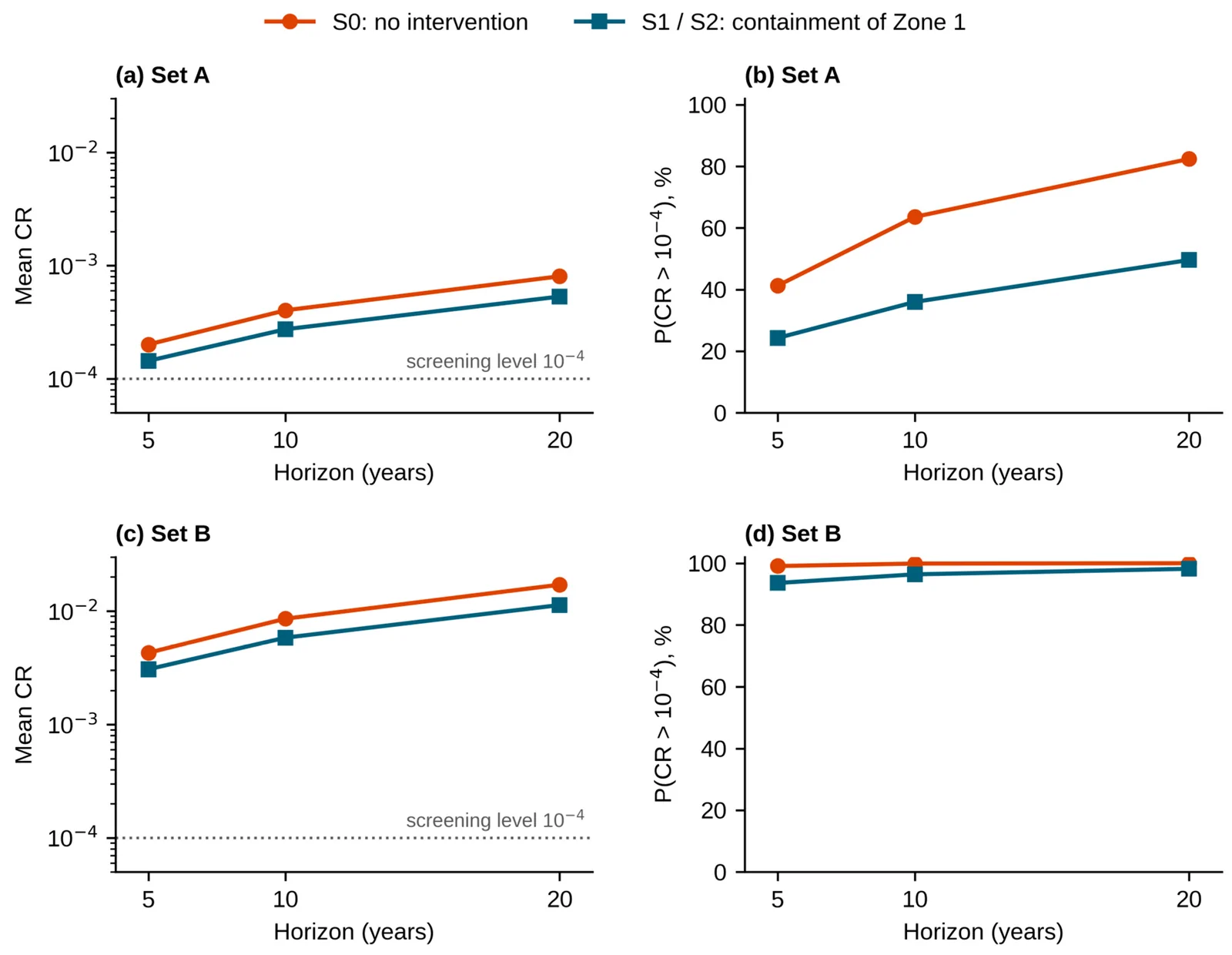
Projected risk under management scenarios at 5-, 10- and 20-year horizons under Set A (**a**,**b**) and Set B (**c**,**d**). (**a**,**c**) Mean carcinogenic risk on a common logarithmic axis; the dotted line marks the 10^−4^ screening level. (**b**,**d**) Probability of exceeding 10^−4^. S0 = no intervention; S1 = engineered containment of Zone 1 (0–10 m); S2 = containment of Zone 1 plus phytostabilization of Zone 2 (10–100 m). S1 and S2 are plotted as a single series because they differ by less than 0.3% at every horizon.

Projected risk under the three management scenarios is given in Table 11 and Figure 6.

Relative mean CR under S1 (S0 = 1.000): 0.716/0.680/0.662 at 5/10/20 yr under Set A; 0.715/0.680/0.661 under Set B. Containment removes roughly a third of mean risk under either parameterization—the proportional benefit of intervention is invariant to the toxicity values, because a slope factor scales all scenarios equally. The revision therefore changes the absolute risk level but not the relative merit of the interventions.

The result for S2 does not depend on the assumption of complete dust suppression. With the dust-reduction efficiency varied from 0 to 100%, mean CR under S2 differed from that under S1 by at most 0.14% under Set A and 0.006% under Set B (20-yr horizon), and the probability of exceeding 10^−4^ was unchanged at the reported precision (Table S6). This follows from the pathway structure: inhalation contributes 0.17% of mean CR under Set A and 0.01% under Set B (Section 3.5), so that complete suppression is an upper bound on the benefit of phytostabilization for the occupational receptor rather than an assumption on which the result depends.

The benefit of containing Zone 1 depends strongly on how the receptor divides time between the zones. At the 20-year horizon under Set A, mean CR under S1 relative to S0 was 0.05, 0.41, 0.66, 0.85 and 1.00 for Zone 1:Zone 2 allocations of 100:0, 75:25, 50:50, 25:75 and 0:100, and P(CR > 10^−4^) under S1 was 5%, 38%, 50%, 56% and 60%, respectively (Table S7). Without intervention, P(CR > 10^−4^) ranged from 60% to 84% under Set A and from 97% to 100% under Set B across these allocations. The screening conclusion therefore does not depend on the allocation, but the effectiveness of containment limited to Zone 1 does: it is substantial only if the worker spends most of the time within 10 m of the heap.

S1 and S2 remain indistinguishable under both sets (<0.3%).

## 4. Discussion

The Discussion distinguishes three kinds of statement. Observed results are properties of the measured concentrations and hold without further assumptions. Model-derived results—exceedance probabilities, sensitivity rankings and scenario outcomes—hold conditionally on the exposure model, the toxicity values and the distributional assumptions. Management implications depend in addition on assumptions that have not been validated at this site, notably occupancy, arsenic bioavailability, the respirable fraction implied by the particulate emission factor, and the choice of receptor; they are stated as conditional on those assumptions.

### 4.1. What a Point Estimate Does Not Convey

Under Set A the probabilistic mean (7.38 × 10^−4^) and the published deterministic estimate (7.0 × 10^−4^), computed at central-tendency exposure values, agree to within about 5% for this dataset, an agreement that depends on the shape of the distributions and should not be generalized to other sites. Deterministic evaluation at central parameter values therefore approximates the distributional mean at this site, and the two frameworks do not disagree about central tendency.

They differ in what they report. The deterministic figure states that a calculated value exceeds 10^−4^; the probabilistic analysis states that 69.9% of plausible exposures do. These are different quantities, and only the second is directly usable in a decision [9] that weighs the cost of intervention against the likelihood of harm. Similarly, the value reported as the site worst case is exceeded by 2.8% of simulated exposures—a figure that cannot be inferred from the point estimate, and that a manager might reasonably regard as either negligible or as requiring action depending on the receptor population size.

Two independent studies that applied both frameworks to a single dataset support the general position. A dual-model comparison across 444 paired soil–crop samples reported that deterministic methods underestimate risk for the most exposed 15–25% of an agricultural population [10]. A study of 740 topsoil samples in Guizhou, China, likewise found mean hazard index and carcinogenic risk values to be higher under the probabilistic approach, and noted that traditional deterministic values may underestimate arsenic risk specifically [33]—the element that dominates risk at the present site. At Shymkent the divergence in the mean is small (5%), which is itself informative: agreement in central tendency does not imply that the point estimate carries the same information as the distribution.

The practical recommendation is narrow and defensible: screening-level assessments at sites with strongly right-skewed concentration distributions should report exceedance probabilities alongside point estimates. This requires no additional sampling—only that the existing data be treated as a distribution [9].

The number of Monte Carlo iterations and the amount of empirical information are separate quantities. The 100,000 iterations fix the numerical precision of each estimate to within 0.16 percentage points, but they cannot add information beyond the 24 samples that define the concentration distributions. Bootstrap intervals, which reflect that limit, span roughly 20 percentage points for the site-wide exceedance probability under Set A and more than an order of magnitude for the ratio between zones (Section 3.3 and Section 3.6). Probabilistic output from a small dataset is therefore most informative where it is robust to resampling—here, the site-wide conclusion under either set of toxicity values—and least informative where it is not, as for the zonal comparison.

### 4.2. Sensitivity to the Toxicity-Value Revision

The 2025 IRIS revision of inorganic arsenic raises mean CR at this site by a factor of 21.2 and mean HI by 4.7. The magnitude follows from the site’s contamination profile: arsenic contributes 99.8% of carcinogenic risk, so the site’s risk estimate is effectively a function of a single toxicity value.

The qualitative conclusion is unaffected. Under both parameterizations the 10^−4^ screening level is exceeded by a majority of simulated exposures (69.9% and 99.3%) and HI = 1 by a large majority (82.1% and 97.3%). The finding of [8] that risk at this site exceeds the screening levels, and that arsenic is its dominant driver, holds under either set of values and is in that sense robust.

The quantitative estimate is not. Under current values the point estimate previously reported as the site worst case falls at the 48.5th percentile—close to the median. This is a property of the revision, not of the earlier calculation [34]: the same concentrations and the same exposure model yield a risk estimate 21 times higher because one toxicity value changed. It does not indicate that contamination or exposure at the site has increased. A decision calibrated to the absolute magnitude of an estimate produced under either set—a remediation trigger, or a comparison against a site assessed at a different date—is therefore calibrated to the toxicity values in force at the time of assessment as much as to site conditions.

The generalizable point concerns comparability rather than correctness. Assessments of arsenic-dominated sites completed before and after January 2025 are not on the same scale. The discrepancy between them is larger than the spatial variability such assessments are designed to resolve. Where risk estimates are compared across sites or over time—as they routinely are in prioritization exercises—the toxicity-value vintage should be reported alongside the estimate [34,35]. Recomputation under current values is inexpensive where the underlying concentration data are published, as they were here. Exceedance probabilities are not, however, equally robust to every source of uncertainty: they were stable under the toxicity revision, sampling uncertainty and the alternative concentration models tested, but remained sensitive to occupancy and arsenic bioavailability, and for any receptor exposed mainly by inhalation they would depend on the particulate emission factor.

### 4.3. Arsenic Governs Precision Under Either Parameterization

Arsenic accounts for 99.8% of mean CR and 91.7% of mean HI, and its concentration shows a rank correlation with total CR (ρ = +0.867) exceeding that of the next-ranked input by a factor of three (Section 3.5). The four exposure parameters that a site-specific survey could realistically refine—exposure duration, ingestion rate, adherence factor and frequency—together account for a small fraction of output variance.

This shapes how additional resources should be allocated. A site-specific occupational exposure survey, which is the conventional response to uncertainty in a screening-level assessment, would reduce output variance marginally. A finer-resolution sampling of arsenic, particularly along the eastern transect where the 10,900 mg·kg^−1^ hotspot was recorded as a single point flanked by markedly lower values, would reduce it substantially. Sensitivity analysis converts an intuition about data gaps into a defensible allocation of survey effort, and this is among the more useful and least-used products of probabilistic assessment.

The dominance of arsenic also reinforces the co-priority argument made in [8] and reported at comparable Pb–Zn smelting sites elsewhere [36,37,38]: at polymetallic Pb–Zn legacy sites, the contaminant with the largest mass burden is not the contaminant that drives risk. Probabilistic treatment widens this divergence rather than narrowing it, because arsenic combines a high slope factor with the heaviest distributional tail among the priority metals, a behaviour with a mineralogical basis: at non-ferrous smelting sites arsenic partitions largely into reducible and residual phases and is hosted in discrete mineral grains rather than dispersed through the soil matrix, producing pronounced heterogeneity at metre scale [39,40].

Comparison with other assessments places these results in a regional and international context. Within Kazakhstan, the only nationwide stochastic assessment of urban soils identified Shymkent as one of the four most contaminated of 22 cities, together with Balkhash, Ust-Kamenogorsk and Ridder, and found carcinogenic risks well above the screening level, driven mainly by ingestion of lead by children [41]. That assessment was based on state monitoring data for Pb, Cd, Cu, Zn and Cr and did not include arsenic. The present results indicate that at a slag reprocessing site within the same city arsenic, rather than lead, governs risk, so that assessments omitting arsenic may understate risk where Pb–Zn metallurgical residues are present. In Kentau, a single-industry town of the same region with a long history of Pb–Zn ore processing, surface soils remain contaminated with Zn, Cd, Pb and Cu [7], which indicates that such residues persist as long-term sources. Internationally, the closest analogue is a probabilistic assessment of arsenic-contaminated allotment-garden soils in the former gold- and arsenic-mining town of Złoty Stok, Poland, where total As ranged from 433 to 1148 mg·kg^−1^ [42]. There, 54% of simulated adult exposures reached a carcinogenic risk of 10^−4^, ingestion and dermal contact accounted for nearly all risk while inhalation was negligible, total As and the soil ingestion rate were the principal drivers, and adjusting ingestion for measured bioaccessibility reduced ingestion-related risk by 89%. The pathway structure and sensitivity ranking found here are consistent with those results despite differences in receptor and exposure assumptions, and the size of the bioaccessibility effect reported there indicates that the lower end of the RBA range examined in Section 3.3 is realistic for mining-affected soils.

### 4.4. Distance from Source Is a Poor Proxy for Risk

The three-tier zonation proposed in [8] prioritizes the 0–10 m perimeter for engineered intervention, the 10–100 m band for phytostabilization, and beyond 100 m for monitored attenuation. The third tier contains no transect samples and was not modelled here, so the present analysis bears on the ordering of the first two tiers only. The ordering follows the intuition of radial decay from a point source.

The present analysis does not confirm that ordering on risk grounds. Mean CR in Zone 2 (10–100 m) is 1.81× that in Zone 1 (0–10 m), and mean HI is 1.72× higher (Section 3.6). The mechanism is visible in the source data: the western transect carries As concentrations of 7610 mg·kg^−1^ at 50 m and 7900 mg·kg^−1^ at 100 m, and Pb of 7850 mg·kg^−1^ at 100 m—a secondary maximum at the outer edge of Zone 2.

This result depends on those two samples. Leave-one-out analysis (Section 3.6, Table 10) shows that excluding either the 50 m or the 100 m western sample reverses the comparison, reducing the Zone 2:Zone 1 mean-As ratio from 1.38 to 0.88 and 0.85 respectively; excluding any of the remaining six samples leaves the ratio at 1.49–1.56. Two of eight samples therefore carry the finding.

This dependence is taken to be informative rather than disqualifying, for two reasons. The two samples are not analytical outliers. They lie on the western transect, which [8] identified as carrying the highest mean concentrations of four priority metals, oriented along the prevailing wind regime, away from the Badam River and toward the A-2 highway and adjacent residential areas. Their values also sit within a broadly elevated arsenic profile along that transect—3890 mg·kg^−1^ at 0 m, 7610 at 50 m and 7900 at 100 m—rather than standing isolated within it. More importantly, the sensitivity is itself the finding: where two samples in eight can reverse the risk ordering between zones, a distance-stratified design with *n* = 8 beyond the perimeter does not resolve the risk surface, and any zonation derived from it—including the ordering proposed in [8] and the reversal suggested here—rests on insufficient support.

What the data do establish is limited. The stratified bootstrap interval for the Zone 2:Zone 1 ratio of mean CR (0.16–9.9) includes values well below and well above unity, so the present design cannot determine whether risk beyond the 10 m perimeter is lower or higher than within it. Pernebayev et al. [8] documented the non-monotonic concentration pattern and declined to fit a radial dispersion model on that basis, while retaining a distance-ordered zonation in its management recommendations. The present results indicate that this zonation is not supported by the risk data, but they equally do not support a reversed ordering.

Zone 1 nevertheless carries the higher exceedance probability (77.5% versus 60.3%). The zones therefore differ in the character of their contamination, not merely in its degree: Zone 1 is uniformly and reliably contaminated; Zone 2 is patchily and severely so. Mean risk responds to the tail; exceedance probability responds to the bulk. A management framework built on either statistic alone will misallocate effort, and the two should be reported together.

The generalizable point is that at sites where deposition is wind-controlled and runoff redistributes coarse fragments along topographic gradients, distance from the source perimeter is a poor proxy for risk. Zonation should follow the measured risk surface, and a sampling design stratified by distance rather than by expected risk will not resolve that surface at reasonable cost. Directional stratification weighted toward the prevailing wind vector, with denser sampling where secondary maxima have already been observed, would support zonation decisions that the present design cannot.

### 4.5. The Receptor Determines Which Intervention Appears Effective

Containment of Zone 1 reduces mean CR to 0.660 of the no-intervention value at the 20-year horizon when time is divided equally between the zones; the reduction ranges from 95% to none as the share of time spent in Zone 1 falls from 100% to zero (Section 3.7). Adding phytostabilization of Zone 2 produces no additional reduction: S1 and S2 differ by less than 0.3% at every horizon (Section 3.7).

This follows directly from the sensitivity analysis. Phytostabilization was modelled as suppression of dust emission, which acts on the inhalation pathway; inhalation contributes 0.17% of worker CR under Set A and 0.01% under Set B under the RAGS default particulate emission factor of 1.36 × 10^9^ m^3^·kg^−1^, and shows negligible rank correlation with output (ρ < 0.01). Within this model, an intervention acting on a pathway that carries almost none of the modelled risk cannot reduce that risk. This is a model outcome for the on-site occupational receptor under the default particulate emission factor, not a general statement about the performance of phytostabilization, whose benefit lies in reducing off-site dust transport and exposure of receptors not modelled here.

This result must not be read as evidence against phytostabilization. It is a statement about the receptor, not the measure. The occupational receptor modelled here stands on the contaminated surface; dust generated at mining and metallurgical sites is nonetheless a documented source of measurable exposure in adjacent settlements [43,44]; ingestion and dermal contact dominate, and a vegetative cover does not remove soil from contact. The receptor that phytostabilization protects is the off-site resident, for whom the transport pathway—wind-driven dispersion of fine particulates—is the exposure pathway. Pernebayev et al. [8] reported Pb at 205 mg·kg^−1^ (12× upper continental crust) at a reference point 1.5 km from the heap, indicating that this transport is operative at the kilometre scale in the prevailing wind regime.

The general implication is methodological. Where a risk assessment is used to compare interventions, the choice of receptor silently determines the ranking. An occupational-only assessment will systematically favour barrier measures over emission-control measures, because barriers act on the pathways that dominate on-site exposure. Site-management frameworks derived from single-receptor assessments therefore encode a preference that is an artefact of scope rather than a property of the interventions. Extension of the present analysis to a residential receptor is the most direct route to resolving this, and is identified as the priority next step (Section 4.6).

### 4.6. Limitations

Single sampling campaign. All concentration data derive from one campaign (24 April 2026). How metal mobility shifts between seasons in this semi-arid setting, where moisture arrives episodically, is therefore not captured; the fitted distributions describe spatial rather than temporal variability.

Total concentrations. No sequential extraction was performed, so the bioavailable fraction is unknown. Total concentrations were used throughout, which is conservative for risk estimation but limits inference about mobility. Fractionation is identified as a priority for the next campaign.

Temporal representativeness. The analysis rests on a single sampling campaign (24 April 2026) at an active reprocessing site. Concentrations may since have changed through continued reprocessing, earthworks or redistribution by wind and runoff, and seasonal variation was not captured; the results describe the site as sampled rather than its current state.

Mercury. Mercury was modelled from total concentrations in soil through the particulate pathways of the RAGS model. Volatilization of elemental mercury and mercury speciation were not considered, although inhalation can be an important route of mercury exposure near metallurgical residues [45] and mercury in settled dust can vary seasonally in semi-arid settings [46]. Because Hg contributes a small share of the modelled hazard index, this omission does not affect the conclusions for the occupational receptor, but it would matter for residential receptors and children.

Analytical quality control. Element-specific analytical quality-control data (certified reference material recoveries, blanks, replicate precision) were not reported in the laboratory test protocols and could not be included. A systematic analytical bias of ±20% altered the probability of exceeding 10^−4^ by about 5 percentage points or less under either parameterization (Table S3), indicating that the conclusions are not sensitive to analytical error of this magnitude.

Sampling depth. Soil was sampled as 0–50 cm depth-integrated composites, and surface-layer (0–5 or 0–10 cm) concentrations, which are most relevant to incidental ingestion and dermal contact, were not measured separately. A twofold difference between surface and depth-integrated concentrations would not change the conclusions under the 2025 toxicity values (Table S3).

Toxicity values. The arsenic slope factors and reference doses under both parameterizations were taken directly from the primary IRIS records. Route-specific inhalation and dermal reference doses required for the hazard index are not issued under the IRIS programme and were obtained by route extrapolation from published compilations, in which such values vary between sources. Carcinogenic risk is unaffected, since it draws only on IRIS slope factors; hazard index values carry this additional uncertainty.

Zone areas and receptor time allocation. Zone boundaries follow the buffer rings of [8], described there as estimated. Combined risk assumes equal receptor time allocation between Zone 1 and Zone 2, an assumption rather than a survey result; repeating the analysis for allocations from 100:0 to 0:100 shows that this assumption governs the estimated benefit of containment but not the screening conclusion (Section 3.7). Results are therefore reported as relative risk ratios rather than absolute exposures, and zonal results are given separately so that alternative weightings can be applied.

Sample size. The fitted distributions rest on 24 samples, and bootstrap intervals for the site-wide exceedance probabilities span roughly 20 percentage points under Set A (Section 3.3); the probabilities should therefore be read to the nearest ten percentage points rather than to one decimal place. Eight samples support the zonal comparison, the direction of the difference is not robust to the exclusion of either western-transect sample, and the stratified bootstrap interval for the Zone 2:Zone 1 ratio spans values on both sides of unity (Section 3.6). The zonal comparison is therefore unresolved by the present design.

Arsenic bioavailability. Carcinogenic risk was computed from total soil arsenic, which is equivalent to assuming a relative bioavailability of unity. The US EPA recommends a default relative bioavailability of 60% for arsenic in soil [28], and in vivo measurements on soils from mining and smelting sites report values well below unity [47]. The estimates reported here are therefore upper bounds with respect to this parameter; applying RBA to the ingestion pathway reduces mean CR by 29% at the default of 0.6 and by 57% at 0.2 (Section 3.3). Under Set B the probability of exceeding 10^−4^ remains above 97% across this range. Under Set A it falls to 62.5% at the default and to 49.5% at 0.2, so that with the superseded toxicity values and low bioavailability, exceedance would be about as likely as not. A site-specific bioaccessibility measurement would therefore matter for conclusions drawn under the superseded values, but not under those currently in force.

Particulate emission factor. The RAGS default particulate emission factor was retained rather than derived site-specifically. This parameter governs the magnitude of the inhalation pathway, and the negligible reduction attributed to phytostabilization in the scenario analysis follows from it. The respirable fraction of the slag, which is the property this factor represents, was not characterized. Aerodynamic or laser-diffraction sizing of the respirable fraction, and a particulate emission factor derived from it, would be required before the S1–S2 comparison could be treated as quantitative rather than indicative; emission from tailings and slag surfaces is governed by the respirable fraction and surface condition rather than by bulk composition, and site-specific factors derived from dispersion measurement differ substantially from generic defaults [48,49].

Intervention efficiencies. Scenario parameters are literature-derived. The scenarios constitute a parametric analysis of the sensitivity of risk to the documented range of intervention performance, not a site-specific engineering forecast. For phytostabilization, the dust-reduction efficiency was varied from 0 to 100% without effect on the conclusions (Section 3.7).

Single receptor. Only an adult occupational receptor was modelled. Extension to a residential receptor would require soil data from residential and sensitive land uses. The nearest housing lies about 250 m west of the heap and about 10,500 people live within 1 km of its centre (Section 2.1), but soil was not sampled at residential locations, so no such analysis is attempted here. As set out in Section 4.5, this scope determines the relative ranking of interventions, and the omission of a residential receptor is the principal limitation on the management conclusions.

## 5. Conclusions

Probabilistic reanalysis of a published dataset from an active Pb–Zn slag reprocessing site shows that the January 2025 IRIS revision of inorganic arsenic changes the quantitative risk estimate far more than any modelling choice: for the same measured concentrations, the mean carcinogenic risk estimated for the adult outdoor worker was 21-fold higher, and the estimated probability of exceeding the upper end of the US EPA risk-management range (10^−4^) increased from about 70% (95% bootstrap interval 59–80%) to over 98%. The qualitative screening conclusion of [8] therefore holds under both sets of toxicity values, whereas estimates made before and after the revision are not comparable; this conclusion is robust to sampling uncertainty and to the alternative concentration models tested, but remains conditional on the occupancy, bioavailability and particulate-emission assumptions of the exposure model. Relative to the published deterministic assessment, the probabilistic analysis changes the central estimate for this dataset by only about 15%, but adds what a point estimate cannot convey: the probability of exceedance and the position of the published values within the distribution. The principal source of uncertainty is the arsenic concentration distribution, which dominates output variance and rests on 24 samples. This small spatial dataset leaves the ordering of risk between the zones unresolved, and the restriction to a single occupational receptor means that the results do not extend to residents or children.

## Figures and Tables

**Table 1:** Relation of the present study to the source assessment [8].

Aspect	Reported in [8]	New in the Present Study
Data	24 transect and 2 reference soil samples, ten elements (ICP-OES)	Same dataset reused; no new sampling
Contamination indices, plant biomonitoring, ecosystem risk	CF, Igeo, EF, PLI, mCd, RI; bulk canopy ratios; PEC/PNEC	Not repeated
Human-health risk	Deterministic point estimates (site mean CR 7.0 × 10^−4^, worst case 4.3 × 10^−3^; HI 4.3 and 26.6)	Full distributions of CR and HI; exceedance probabilities; position of the point estimates within the distributions
Toxicity values	Values in force before 2025	Reassessment under the January 2025 IRIS arsenic values; effect of the revision separated from the effect of probabilistic treatment
Uncertainty	Not quantified	Sensitivity of CR and HI to all inputs; bootstrap sampling uncertainty; alternative distributions, inter-metal dependence and analytical error
Zonation	Three-tier zonation by distance from the heap	Tested against modelled risk; ordering of zones not resolved by the data
Interventions	Qualitative three-tier framework	Time-resolved scenario projections with sensitivity to efficiency assumptions
Principal conclusion	Arsenic dominates human-health risk; risk exceeds the screening level	Qualitative conclusion holds under both sets of toxicity values; quantitative estimate changes 21-fold because of the toxicity values alone

**Table 2:** Fitted distribution parameters for the 24 transect samples.

Metal	GM	GSD	AM	Min	Max	Shapiro–Wilk p (log)	Model
Pb	776.9	3.86	1754	72.6	9350	0.339	lognormal
Zn	822.3	3.51	1254	7.79	2870	<0.001	empirical
Cd	22.4	3.65	53.7	3.51	290	0.108	lognormal
Cu	405.5	2.68	684	80.1	4230	0.638	lognormal
As	658.2	3.94	1753	95.2	10,900	0.176	lognormal
Hg	1.19	6.31	8.0	0.12	47.4	<0.001	empirical
Ni	10.2	1.94	12.4	2.31	27.1	0.373	lognormal
Cr	6.88	2.22	9.4	1.79	36.9	0.955	lognormal

GM—geometric mean; GSD—geometric standard deviation; AM—arithmetic mean. All concentrations in mg·kg^−1^.

**Table 3:** Stochastic exposure parameters, adult outdoor-worker scenario.

Parameter	Distribution	Parameters	Central Value
Body weight (BW)	Normal, truncated to 40–130	μ = 70, σ = 12 kg	70 kg
Soil ingestion rate (IngR)	Lognormal	GM = 50, GSD = 2.0 mg·d^−1^	50 mg·d^−1^
Inhalation rate (InhR)	Normal, truncated to 8–35	μ = 20, σ = 3 m^3^·d^−1^	20 m^3^·d^−1^
Exposed skin area (SA)	Normal, truncated to 1500–5500	μ = 3300, σ = 660 cm^2^	3300 cm^2^
Adherence factor (AF)	Lognormal	GM = 0.2, GSD = 2.0 mg·cm^−2^	0.2 mg·cm^−2^
Exposure frequency (EF)	Triangular	180/250/350 d·yr^−1^	250 d·yr^−1^
Exposure duration (ED)	Triangular	1/25/40 yr	25 yr
Particulate emission factor (PEF)	Fixed	1.36 × 10^9^ m^3^·kg^−1^	—
Averaging time, carcinogenic	Fixed	70 × 365 d	—
Averaging time, non-carcinogenic	Fixed	ED × 365 d	—

**Table 4:** Toxicity values applied under the two parameterizations.

Parameter (Inorganic Arsenic)	Set A	Set B	Basis of Set B
Oral slope factor, per mg·kg^−1^·d^−1^	1.5	32	Combined bladder (17.6) and lung (21.3) cancer slope factors; Bayesian hierarchical dose–response meta-analysis, logistic-power model; 11 bladder and 6 lung cancer studies. Updated 13 January 2025
Dermal slope factor, per mg·kg^−1^·d^−1^	1.5	32	Oral slope factor with GI absorption = 1.0
Inhalation slope factor, per mg·kg^−1^·d^−1^	15.1	15.1	Derived from IUR = 4.3 × 10^−3^ per µg·m^−3^ (1995), not revised in 2025
Oral RfD, mg·kg^−1^·d^−1^	3.0 × 10^−4^	6.0 × 10^−5^	Endocrine and cardiovascular systems; ischemic heart disease and type 2 diabetes; UF_C = 3; confidence medium/high. Rounded from osRfDs of 5.8 × 10^−5^ (type 2 diabetes) and 5.7 × 10^−5^ (ischemic heart disease). Updated 13 January 2025
Slope factors and RfDs, other seven metals	unchanged between sets	unchanged	IRIS

**Table 5:** Distributions of CR and HI under both parameterizations.

Statistic	CR, Set A	CR, Set B	HI, Set A	HI, Set B
Mean	7.38 × 10^−4^	1.57 × 10^−2^	8.03	37.4
5th percentile	1.78 × 10^−5^	3.63 × 10^−4^	0.439	1.41
25th percentile	7.91 × 10^−5^	1.66 × 10^−3^	1.35	5.04
Median	2.26 × 10^−4^	4.77 × 10^−3^	3.12	13.0
75th percentile	6.38 × 10^−4^	1.35 × 10^−2^	7.58	34.2
95th percentile	2.86 × 10^−3^	6.08 × 10^−2^	29.2	141
99th percentile	8.20 × 10^−3^	1.75 × 10^−1^	78.3	386
Maximum	3.44 × 10^−1^	7.34	3040	15,200

**Table 6:** Exceedance probabilities against screening levels.

Threshold	Set A	Set B
CR > 10^−6^	99.98%	100.0%
CR > 10^−5^	97.8%	100.0%
CR > 10^−4^	69.9%	99.3%
CR > 10^−3^	16.8%	84.0%
HI > 1	82.1%	97.3%
HI > 4	42.4%	80.1%
HI > 10	19.1%	57.4%

**Table 7:** Position of the published point estimates within the simulated distributions.

Published Estimate	Value	Percentile, Set A	Percentile, Set B
Site-mean CR	7.0 × 10^−4^	76.9	11.0
Worst-case CR	4.3 × 10^−3^	97.2	47.3
Site-mean HI	4.3	59.7	21.5
Worst-case HI	26.6	94.3	69.2

**Table 8:** Sensitivity of total carcinogenic risk to model inputs (Spearman rank correlation, 100,000 iterations). The seven metal concentrations other than As (|ρ| ≤ 0.005) are not listed here; they are given, together with the full ranking for CR and HI under both parameterizations, in Table S5.

Input	ρ
As concentration	+0.867
Soil ingestion rate (IngR)	+0.292
Exposure duration (ED)	+0.268
Adherence factor (AF)	+0.133
Body weight (BW)	−0.109
Exposure frequency (EF)	+0.084
Exposed skin area (SA)	+0.040
Inhalation rate (InhR)	−0.004

**Table 9:** Zonal risk, no-intervention scenario, 20-yr horizon.

Zone	*n*	Mean As (mg·kg^−1^)	Max As	Mean CR	P(CR > 10^−4^)	Mean HI
Zone 1 (0–10 m)	16	1557	10,900	5.72 × 10^−4^	77.5%	7.0
Zone 2 (10–100 m)	8	2144	7900	1.04 × 10^−3^	60.3%	12.0

**Table 10:** Leave-one-out stability of the Zone 2:Zone 1 mean-As ratio (Zone 1 mean As = 1557 mg·kg^−1^, *n* = 16; full Zone 2 ratio = 1.38).

Sample Excluded	Transect	Distance (m)	As in Sample	Zone 2 Mean As	Ratio
247	South	50	193	2423	1.56
248	South	100	97.6	2436	1.56
253	North	50	183	2424	1.56
254	North	100	95.2	2437	1.56
259	East	50	194	2423	1.56
260	East	100	879	2325	1.49
265	West	50	7610	1363	0.88
266	West	100	7900	1322	0.85

**Table 11:** Scenario projections under both parameterizations.

**Set A—Toxicity Values of the Source Assessment**			
**T (yr)**	**Scenario**	**Mean CR**	**P(CR > 10^−4^)**
5	S0	2.01 × 10^−4^	41.3%
5	S1/S2	1.44 × 10^−4^	24.3%
10	S0	4.03 × 10^−4^	63.6%
10	S1/S2	2.74 × 10^−4^	36.0%
20	S0	8.05 × 10^−4^	82.4%
20	S1/S2	5.33 × 10^−4^	49.6%
**Set B—2025 IRIS values**			
**T (yr)**	**Scenario**	**Mean CR**	**P(CR > 10^−4^)**
5	S0	4.28 × 10^−3^	99.1%
5	S1/S2	3.06 × 10^−3^	93.6%
10	S0	8.56 × 10^−3^	99.9%
10	S1/S2	5.82 × 10^−3^	96.4%
20	S0	1.71 × 10^−2^	100.0%
20	S1/S2	1.13 × 10^−2^	98.2%

## Data Availability

The source geochemical data analysed in this study are openly available as Supplementary Table S1 of reference [8] at https://doi.org/10.3390/su18136742. The simulation code and its console output are provided in the Supplementary Materials. Further inquiries can be directed to the corresponding authors.

## Supplementary Materials

The following supporting information can be downloaded at: https://www.mdpi.com/article/10.3390/toxics14100894/s1, Figure S1: normal quantile–quantile plots of the log-transformed concentrations of all eight metals, with Shapiro–Wilk *p* values; Figure S2: measured concentrations along the four transects as multiples of the Kazakhstan maximum permissible concentration; Table S2: bootstrap uncertainty due to the limited number of samples, site-wide and by zone; Table S3: sensitivity to analytical error and sampling depth; Table S4: goodness of fit of alternative distributions, risk under alternative concentration models and dependence structures, and kernel width; Table S5: rank-correlation sensitivity of CR and HI to all stochastic inputs; Table S6: sensitivity of scenario S2 to the dust-reduction efficiency of phytostabilization; Table S7: sensitivity to the allocation of time between zones; Table S8: sensitivity to the relative bioavailability of arsenic. Script S1: ‘S1_reproduce_all.py’, which reproduces every result of the primary analysis from the published source dataset (fixed random seed 20260827); File S2: ‘S2_expected_output.txt’, the console output of Script S1; File S3: ‘requirements.txt’, the library versions used; Script S4: ‘S4_revision_block1.py’, ‘S4_revision_block2.py’ and ‘S4_revision_block3.py’, which reproduce the analyses in Tables S2–S8 and Figure S2, and ‘S4_revision_figures.py’, which reproduces Figure 3, Figure 4, Figure 5 and Figure 6; File S5: ‘S5_revision_output.txt’, the console output of Script S4. The scripts require Supplementary Table S1 of [8], publicly available under a CC BY licence at https://doi.org/10.3390/su18136742; that dataset is not duplicated here.

## Abbreviations

The following abbreviations are used in this manuscript:

ABS	Dermal absorption factor
AF	Adherence factor
AT	Averaging time
BW	Body weight
CDI	Chronic daily intake
CR	Carcinogenic risk
ED	Exposure duration
EF	Exposure frequency
GM	Geometric mean
GSD	Geometric standard deviation
HI	Hazard index
ICP-OES	Inductively coupled plasma optical emission spectrometry
IngR	Soil ingestion rate
InhR	Inhalation rate
IRIS	Integrated Risk Information System
IUR	Inhalation unit risk
MPC	Maximum permissible concentration
PEF	Particulate emission factor
RAGS	Risk Assessment Guidance for Superfund
RfD	Reference dose
SA	Exposed skin area
SF	Slope factor
US EPA	United States Environmental Protection Agency
