# Supplementary material for: Exceedance Probability and Toxicity-Value Sensitivity in Health-Risk Assessment of a Pb–Zn Slag Reprocessing Site Under the 2025 IRIS Arsenic Values
Source: Toxics. 2026 Oct 7;14(10):894. doi: 10.3390/toxics14100894 (PMC13709863; doi:10.3390/toxics14100894)
Supplement: Supplementary file 1 [file toxics-14-00894-s001.zip › toxics-4581504_Supplementary_R1.pdf]

Supplementary Materials

Exceedance Probability and Toxicity-Value Sensitivity in Health-Risk Assessment of a Pb–Zn Slag Reprocessing Site under the 2025 IRIS Arsenic Values

Set A: toxicity values applied in the source assessment [8]; Set B: values established by the January 2025 IRIS revision of inorganic arsenic. CR, carcinogenic risk; HI, hazard index. Unless stated otherwise, results refer to the adult outdoor worker, all 24 transect samples and 100 000 Monte Carlo iterations. All values are reproduced by Scripts S1 and S4 (see the list at the end of this document).

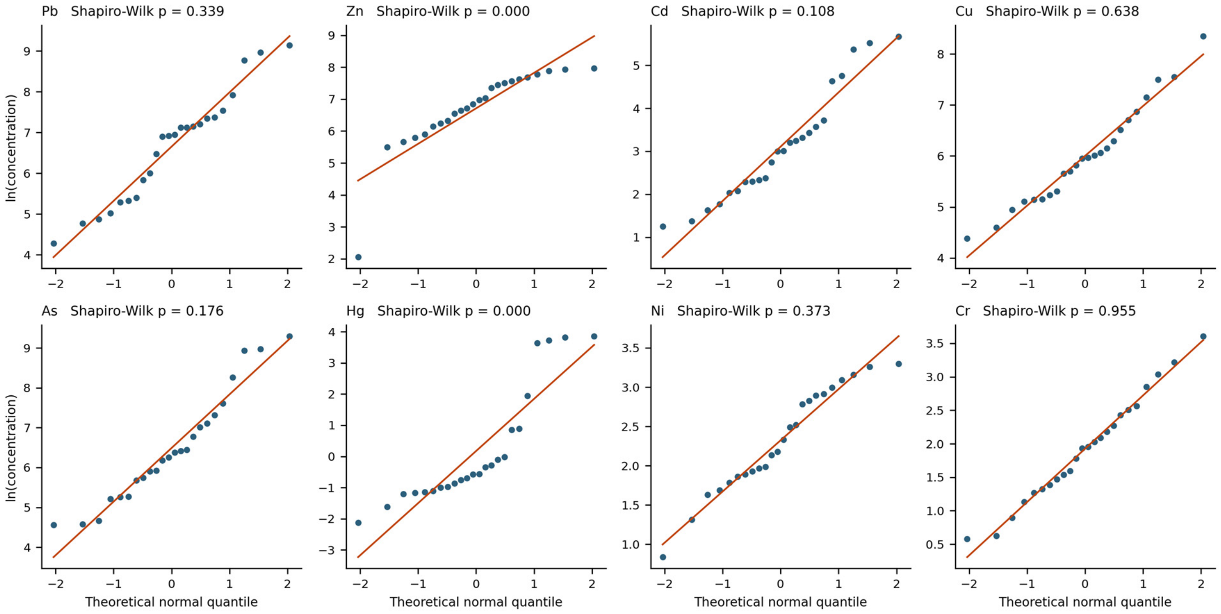

Figure S1. Normal quantile–quantile plots of the log-transformed concentrations of the eight metals, with Shapiro–Wilk p values (Section 2.3).

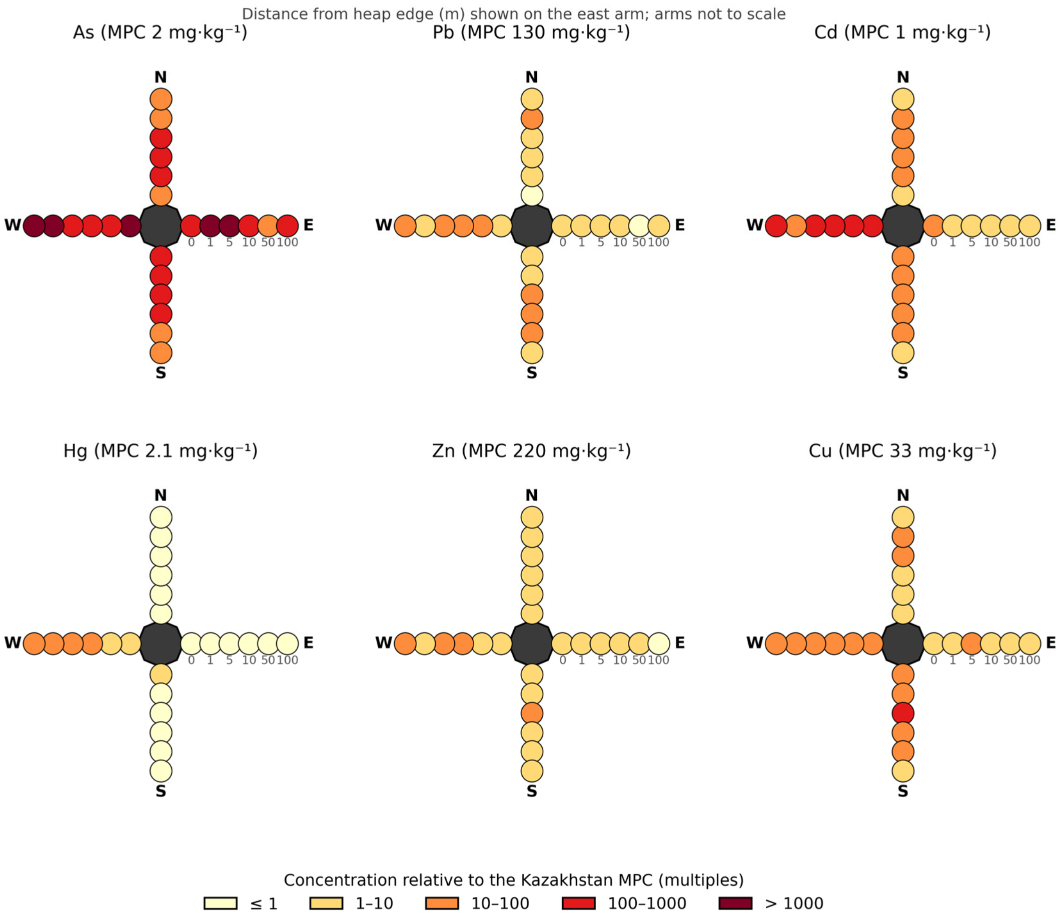

Figure S2. Measured concentrations of As, Pb, Cd, Hg, Zn and Cu at the 24 sampling points along the four transects, expressed as multiples of the Kazakhstan maximum permissible concentration (MPC) applied in [8]. Points are placed at their transect and distance from the heap edge (0, 1, 5, 10, 50 and 100 m); arm lengths are schematic and not to scale. Interpolated surfaces were not produced because the sectors between transects are unsampled (Section 3.1).

Table S2. Uncertainty due to the limited number of samples: nonparametric bootstrap (1000 replicates × 10 000 iterations). Point estimates are from the primary analysis.

Metric	Point estimate	2.5th percentile	Median	97.5th percentile
Set A: mean CR	7.38×10^{-4}	2.98×10^{-4}	7.22×10^{-4}	1.64×10^{-3}
Set A: P(CR > 10^{-4}), %	69.9	59.1	70.9	80.4
Set A: mean HI	8.03	3.5	7.94	17.3
Set A: P(HI > 1), %	82.1	72.5	82.6	90.1
Set B: mean CR	1.57×10^{-2}	6.33×10^{-3}	1.54×10^{-2}	3.49×10^{-2}
Set B: P(CR > 10^{-4}), %	99.3	98.4	99.4	99.9
Set B: mean HI	37.4	15.3	36.5	82.9
Set B: P(HI > 1), %	97.3	94.5	97.5	99.2
As geometric mean, mg·kg^{-1}	658	397	–	1115
As geometric standard deviation	3.94	2.59	–	5.23
Zone 2 : Zone 1 ratio of mean CR (stratified; Set A, 20 yr)	1.81	0.16	1.84	9.91
Zone 2 : Zone 1 ratio of mean CR (stratified; Set B, 20 yr)	1.81	0.16	1.85	9.93

In all 1000 replicates P(CR > 10^{-4}) and P(HI > 1) exceeded 50% under both sets. Zone 2 exceeded Zone 1 in 67.6% of stratified replicates. The Monte Carlo standard error of an exceedance probability at 100 000 iterations is at most 0.16 percentage points.

Table S3. Sensitivity to analytical error and to sampling depth. Concentrations of all eight metals were scaled uniformly or perturbed by random multiplicative error before the distributions were refitted.

Perturbation	Set A P(CR > 10^{-4}), %	Set A P(HI > 1), %	Set B P(CR > 10^{-4}), %	Set B P(HI > 1), %
None (primary analysis)	69.9	82.1	99.3	97.3
Analytical bias ×0.8	64.8	76.8	98.9	95.9
Analytical bias ×1.2	73.9	85.9	99.5	98.1
Random error, 10% RSD (95% range of 200 replicates)	68.8–71.3	81.0–83.0	99.0–99.5	96.8–97.6
Random error, 20% RSD (95% range of 200 replicates)	67.4–71.9	80.0–83.5	98.9–99.5	96.2–97.8
Surface / depth-integrated ratio 0.5	53.1	63.8	97.8	91.4
Surface / depth-integrated ratio 2.0	83.4	93.6	99.8	99.4

Table S4. Robustness of the concentration model. (a) Goodness of fit of alternative marginal distributions (maximum likelihood, location fixed at 0); (b) risk under alternative concentration models and dependence structures; (c) kernel width used for the empirical resampling of Zn and Hg.

(a) Goodness of fit

Metal	Lognormal AIC	Gamma ΔAIC	Weibull ΔAIC	KS p (lognormal / gamma / Weibull)
As	398	8.54	6.24	0.70 / 0.16 / 0.37
Pb	405	3.91	2.90	0.54 / 0.47 / 0.67
Cd	233	8.03	6.26	0.73 / 0.20 / 0.41
Cu	359	6.60	6.67	0.93 / 0.37 / 0.53
Ni	163 (ΔAIC 0.45)	0 (lowest)	0.39	0.71 / 0.67 / 0.61
Cr	153	2.94	4.33	0.99 / 0.90 / 0.94

ΔAIC relative to the lowest-AIC model for each metal. Kolmogorov–Smirnov p values are conservative because parameters were estimated from the same data. Zn and Hg are modelled by empirical resampling (Shapiro–Wilk on log values, $p < 0.001$).

(b) Alternative concentration models and dependence structures

Model	Set A mean CR	Set A P(CR > 10^{-4}), %	Set A P(HI > 1), %	Set B P(CR > 10^{-4}), %	Set B P(HI > 1), %
Primary: lognormal/empirical, independent	7.38×10^{-4}	69.9	82.1	99.3	97.3
As gamma, others as primary	7.77×10^{-4}	71.7	81.7	95.5	93.1
All six parametric metals gamma	7.70×10^{-4}	71.9	82.6	95.6	93.4
As Weibull, others as primary	7.32×10^{-4}	69.1	80.0	95.5	92.8
All six parametric metals Weibull	7.33×10^{-4}	68.9	80.4	95.5	92.9
Independent empirical resampling, all metals	7.75×10^{-4}	67.7	81.5	99.8	98.8
Joint row resampling, no kernel	7.68×10^{-4}	68.1	78.1	99.8	98.3
Joint row resampling, kernel σ = 0.15	7.78×10^{-4}	68.1	78.1	99.8	98.2
Gaussian copula, primary marginals	7.38×10^{-4}	70.0	79.7	99.3	96.5

Joint row resampling and the Gaussian copula preserve the observed inter-metal dependence (Spearman ρ = 0.80–0.90 among Pb, Zn, Cd, Cu and Ni; As–others 0.17–0.32; As–Hg 0.47).

(c) Kernel width for Zn and Hg

σ	Set A P(CR > 10^{-4}), %	Set A P(HI > 1), %	Set B P(CR > 10^{-4}), %	Set B P(HI > 1), %
0	69.9	82.1	99.3	97.3
0.05	69.9	82.1	99.3	97.3
0.15 (primary)	69.9	82.1	99.3	97.3
0.30	69.9	82.2	99.3	97.3

Table S5. Spearman rank correlation between each of the fifteen stochastic inputs and total CR and HI, under both parameterizations, with the approximate contribution to output variance (squared correlations normalised to 100%).

Input	ρ CR, A	ρ HI, A	ρ CR, B	ρ HI, B	Var. CR A, %	Var. HI A, %	Var. CR B, %	Var. HI B, %
As concentration	+0.867	+0.852	+0.868	+0.904	79.3	79.0	79.6	85.4
Soil ingestion rate	+0.292	+0.277	+0.291	+0.243	9.0	8.4	9.0	6.2
Exposure duration	+0.268	−0.001	+0.266	−0.001	7.6	0.0	7.5	0.0
Adherence factor	+0.133	+0.242	+0.132	+0.227	1.9	6.4	1.8	5.4
Body weight	−0.109	−0.132	−0.109	−0.121	1.3	1.9	1.2	1.5
Exposure frequency	+0.084	+0.101	+0.083	+0.092	0.7	1.1	0.7	0.9
Exposed skin area	+0.040	+0.071	+0.039	+0.066	0.2	0.6	0.2	0.5
Pb concentration	+0.000	+0.153	+0.000	+0.044	0.0	2.5	0.0	0.2
Cd concentration	+0.001	+0.026	+0.001	+0.007	0.0	0.1	0.0	0.0
Hg concentration	−0.005	+0.003	−0.005	−0.004	0.0	0.0	0.0	0.0
Inhalation rate	−0.004	−0.005	−0.004	−0.006	0.0	0.0	0.0	0.0
Cu concentration	−0.002	+0.001	−0.002	−0.001	0.0	0.0	0.0	0.0
Cr concentration	+0.002	−0.000	−0.003	−0.001	0.0	0.0	0.0	0.0
Ni concentration	−0.001	−0.003	−0.001	−0.002	0.0	0.0	0.0	0.0
Zn concentration	−0.000	+0.001	+0.000	+0.000	0.0	0.0	0.0	0.0

Pathway shares of mean CR under Set A: ingestion 71.4%, dermal contact 28.4%, inhalation 0.17% (Set B: 71.5%, 28.5%, 0.01%). Pathway shares of mean HI under Set A: ingestion 51.9%, dermal contact 48.1%, inhalation 0.03%.

Table S6. Sensitivity of scenario S2 (containment of Zone 1 plus phytostabilization of Zone 2) to the dust-reduction efficiency of phytostabilization. Difference in mean CR relative to S1 (containment of Zone 1 only).

Efficiency	5 yr, Set A	10 yr, Set A	20 yr, Set A	5 yr, Set B	10 yr, Set B	20 yr, Set B
0%	0	0	0	0	0	0
25%	−0.012%	−0.026%	−0.034%	−0.001%	−0.001%	−0.002%
50%	−0.023%	−0.053%	−0.069%	−0.001%	−0.002%	−0.003%
75%	−0.035%	−0.079%	−0.103%	−0.002%	−0.004%	−0.005%
100% (primary)	−0.046%	−0.106%	−0.138%	−0.002%	−0.005%	−0.006%

P(CR > 10^{-4}) under S2 equalled that under S1 to one decimal place at every efficiency and horizon (Set A: 24.3%, 36.0%, 49.6% at 5, 10, 20 yr; Set B: 93.6%, 96.4%, 98.2%).

Table S7. Sensitivity of the scenario results to the allocation of the worker's time between Zone 1 (0–10 m) and Zone 2 (10–100 m), 20-yr horizon. S0, no intervention; S1, containment of Zone 1.

Zone 1 : Zone 2	Set	S0 mean CR	S0 P(CR > 10^{-4}), %	S1 mean CR	S1 P(CR > 10^{-4}), %	S1 / S0 mean CR
100:0	A	5.72×10^{-4}	77.5	2.86×10^{-5}	5.2	0.05
100:0	B	1.22×10^{-2}	99.9	6.08×10^{-4}	78.7	0.05
75:25	A	6.89×10^{-4}	83.7	2.81×10^{-4}	38.2	0.41
75:25	B	1.46×10^{-2}	100.0	5.97×10^{-3}	97.5	0.41
50:50 (primary)	A	8.05×10^{-4}	82.4	5.33×10^{-4}	49.6	0.66
50:50 (primary)	B	1.71×10^{-2}	100.0	1.13×10^{-2}	98.2	0.66
25:75	A	9.22×10^{-4}	76.9	7.86×10^{-4}	56.1	0.85
25:75	B	1.96×10^{-2}	99.9	1.67×10^{-2}	98.0	0.85
0:100	A	1.04×10^{-3}	60.3	1.04×10^{-3}	60.3	1.00
0:100	B	2.21×10^{-2}	96.8	2.21×10^{-2}	96.8	1.00

Table S8. Sensitivity to the relative oral bioavailability (RBA) of arsenic, applied to the ingestion pathway.

RBA	Set A mean CR	Set A P(CR > 10^{-4}), %	Set A P(HI > 1), %	Set B mean CR	Set B P(CR > 10^{-4}), %	Set B P(HI > 1), %
0.2	3.17×10^{-4}	49.5	72.3	6.71×10^{-3}	97.2	93.8
0.4	4.22×10^{-4}	57.1	75.8	8.95×10^{-3}	98.3	95.3
0.6 (US EPA default)	5.27×10^{-4}	62.5	78.3	1.12×10^{-2}	98.7	96.2
0.8	6.32×10^{-4}	66.6	80.4	1.34×10^{-2}	99.1	96.8
1.0 (primary)	7.38×10^{-4}	69.9	82.1	1.57×10^{-2}	99.3	97.3

Scripts and output files

Script S1 (S1_reproduce_all.py) reproduces every result of the primary analysis; File S2 is its console output; File S3 lists the library versions. Script S4 (S4_revision_block1.py, S4_revision_block2.py, S4_revision_block3.py) reproduces the analyses added in revision: block 1 — inter-metal correlation, dependence structures, alternative distributions, kernel width and bootstrap (Tables S2 and S4); block 2 — sensitivity to all inputs, arsenic bioavailability, zone time allocation and phytostabilization efficiency (Tables S5–S8); block 3 — analytical error, sampling depth and the stratified zonal bootstrap (Tables S2 and S3); S4_revision_figures.py reproduces Figures 3–6 of the article. Their console outputs are provided as File S5. All scripts require Supplementary Table S1 of [8] (https://doi.org/10.3390/su18136742) in the working directory.
